# The *Chlamydia* Type III Secretion System C-ring Engages a Chaperone-Effector Protein Complex

**DOI:** 10.1371/journal.ppat.1000579

**Published:** 2009-09-11

**Authors:** Kris E. Spaeth, Yi-Shan Chen, Raphael H. Valdivia

**Affiliations:** Department of Molecular Genetics and Microbiology and Center for Microbial Pathogenesis, Duke University Medical Center, Durham, North Carolina, United States of America; The Rockefeller University, United States of America

## Abstract

In Gram-negative bacterial pathogens, specialized chaperones bind to secreted effector proteins and maintain them in a partially unfolded form competent for translocation by type III secretion systems/injectisomes. How diverse sets of effector-chaperone complexes are recognized by injectisomes is unclear. Here we describe a new mechanism of effector-chaperone recognition by the *Chlamydia* injectisome, a unique and ancestral line of these evolutionarily conserved secretion systems. By yeast two-hybrid analysis we identified networks of *Chlamydia*-specific proteins that interacted with the basal structure of the injectisome, including two hubs of protein-protein interactions that linked known secreted effector proteins to CdsQ, the putative cytoplasmic C-ring component of the secretion apparatus. One of these protein-interaction hubs is defined by Ct260/Mcsc (Multiple cargo secretion chaperone). Mcsc binds to and stabilizes at least two secreted hydrophobic proteins, Cap1 and Ct618, that localize to the membrane of the pathogenic vacuole (“inclusion”). The resulting complexes bind to CdsQ, suggesting that in *Chlamydia*, the C-ring of the injectisome mediates the recognition of a subset of inclusion membrane proteins in complex with their chaperone. The selective recognition of inclusion membrane proteins by chaperones may provide a mechanism to co-ordinate the translocation of subsets of inclusion membrane proteins at different stages in infection.

## Author Summary

The obligate intracellular bacteria *Chlamydia trachomatis* is a common sexually transmitted pathogen and the leading cause of preventable blindness worldwide. *Chlamydia* co-opts host cells by secreting virulence factors directly into target cells through a multi-protein complex termed a type III secretion system or “injectisome”. The lack of a system for molecular genetic manipulation in these pathogens has hindered our understanding of how the *Chlamydia* injectisome is assembled and how secreted factors are recognized and translocated. In this study, a yeast two-hybrid approach was used to identify networks of *Chlamydia* proteins that interact with components of the secretion apparatus. CdsQ, a conserved structural component predicted to be at the base of the injectisome, interacted with multiple proteins, including a new chaperone that binds to and stabilizes secretory cargo destined for the membrane of the pathogenic vacuole. These results suggest that the base of the secretion apparatus serves as a docking site for a chaperone and a subset of chaperone-cargo complexes. Because the chlamydial injectisome represents a unique and ancestral lineage of these virulence-associated secretion systems, findings made in *Chlamydia* should provide unique insights as to how effector proteins are recognized and stabilized, and how a hierarchy of virulence protein secretion may be established by Gram-negative bacterial pathogens.

## Introduction

The obligate, intracellular bacterium *Chlamydia trachomatis* infects the epithelium of the genital tract and conjunctivae, causing a wide range of ailments including the blinding disease trachoma, conjunctivitis, salpingitis, pelvic inflammatory disease and infertility [1]. *Chlamydiae* display an elaborate life cycle beginning with the attachment of an elementary body (EB), the infectious form of the bacteria, to the surface of epithelial cells [2]. Shortly after invasion, EBs differentiate into reticulate bodies (RBs). The RB-containing vacuole is rapidly segregated from normal endosomal maturation pathways to generate a membrane-bound “inclusion” [3]. As the inclusion expands, chlamydial replication becomes asynchronous to yield both RBs and EBs. Eventually, most of the cytoplasmic space of the host cell is occupied by the inclusion and EBs exit the host cell to infect adjacent cells [4].

All *Chlamydiae* code for the core components of a type III secretion (T3S) apparatus, a protein transport system used by Gram-negative bacteria to translocate effector proteins directly into host cells [5]. T3S systems are macromolecular structures composed of 20–35 proteins that are often referred to as “injectisomes” due to their resemblance to an injection needle [6]. *Chlamydia* injectisome components are present at all stages of infection and needle-like structures have been observed on the surface of EBs and at the sites of RB attachment to inclusion membranes [7],[8],[9], suggesting that this secretion system is functional. Putative chlamydial targets of T3S have been identified by their ability to be secreted by *Shigella*, *Yersinia* or *Salmonella* injectisomes [10],[11],[12],[13]. These T3S substrates include >25 soluble proteins and a large family of ∼40–50 integral membrane proteins of unknown function that localize to the inclusion membrane (Incs) [8],[10],[11],[12],[13]. At least one of these effectors, Tarp, is translocated during EB invasion of epithelial cells [10]. T3S substrates are likely translocated in a hierarchical fashion to manipulate specific cellular functions at distinct stages of infection [14].

Injectisomes belong to at least seven distinct families [15] of which three (Ysc, SPI-1 and SPI-2) are predominantly found in free-living pathogens of animals and two are more common in plant pathogens (Hrp1 and Hrp2). The remaining injectisome families are limited to the *Chlamydiae* phylum and the *Rhizobiale* order [6]. In the *Chlamydiales*, the genes encoding the T3S apparatus are scattered in small genomic islands [5],[16]. Remarkably, the content and synteny of these gene clusters is largely conserved among the *Chlamydiaceae*, suggesting that even though members of this phylum diverged over 700 million years ago [17], the genetic blueprint for the assembly of this translocation system has remained largely intact. These findings, combined with the lack of evidence for robust lateral gene transfer of T3S genes in *Chlamydiae*, support the hypothesis that the chlamydial T3S system is the closest to the primordial injectisome [18].

A transcriptional analysis of genes encoding chlamydial injectisome components revealed 10 operons containing 37 genes [16]. Recent work in *C. pneumoniae* indicated that interactions among components of the basal structure of the secretion apparatus are evolutionarily conserved [19]. For example, the putative C-ring component, CdsQ, interacts with the cytoplasmic component CdsL, mimicking similar interactions between YscL and YscQ in *Yersinia* [19],[20]. Although many components of the chlamydial injectisome basal structure are conserved, the needle and needle tip components are not [21]. The identification of needle component, CdsF, and its chaperones CdsE and CdsG, required more sophisticated bioinformatic approaches [22],[23]. Similarly, two sets of translocation pore components, CopB/CopD and CopB2/CopD2 (CT578/CT579 and CT861/CT860, respectively) were identified based on their close linkage to genes encoding secretion chaperones and their similarity in protein topology to the *Yersinia* translocators YopB and YopD [24],[25],[26].

The lack of a system for genetic manipulation in *Chlamydia* and the observation that chlamydial proteins cannot functionally substitute for orthologous T3S components in other bacterial systems [24] has limited a functional analysis of this ancestral secretion system. As a result, little is known as to how the *Chlamydia* injectisome is assembled, regulated or how the translocation of effector proteins is temporally and spatially controlled. Here, we applied yeast two-hybrid (Y2H) technology [27] to identify new core components, regulators and secretory cargo of the chlamydial injectisome. In this manner, we identified two protein-protein interaction nodes linking multiple effector proteins to CdsQ, the putative C-ring component at the base of the injectisome. We provide evidence that one of these protein interaction hubs is a chaperone for a subset of proteins destined for transport to the inclusion membrane and that CdsQ interacts with this chaperone alone and in complex with effector proteins. We propose that the C-ring of the chlamydial injectisome acts as a platform for the recognition and engagement of chaperones complexed to secretory cargo.

## Results

### Identification of *Chlamydia* protein-protein interaction networks by yeast two-hybrid analysis

**Figure 1:**
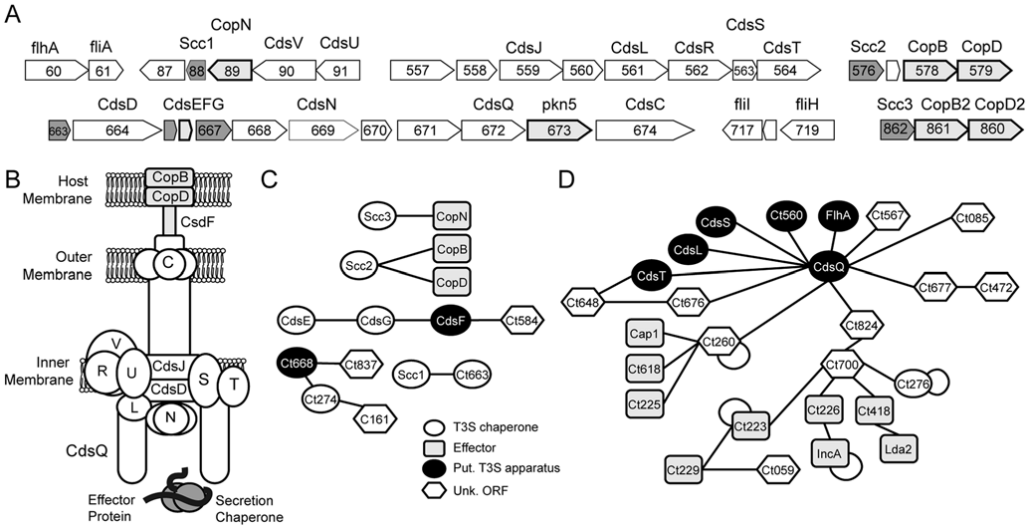
Yeast two-hybrid analysis defines hubs of protein-protein interactions linking the *Chlamydia* injectisome to secreted effectors. (A). Operon arrangement of conserved component of the *C. trachomatis* type III secretion system (T3SS) [16]. Light grey: secreted proteins, Dark grey: T3S chaperones (B). *Proposed architecture of the Chlamydia injectisome*. Assignments were made based on protein interactions conserved in other bacterial T3SS [6] and as proposed in [5]. Cds: contact dependent secretion protein, Cop: chlamydial outer protein. (C). *Interactions between C. trachomatis T3S chaperones and their putative cargo*. Protein-protein interactions were identified based on reciprocal YTH interactions. (D). *Identification of interactions among core components of the C. trachomatis T3S apparatus, chlamydial proteins of unknown function and known effector proteins*. Networks of putative interacting proteins were identified by Y2H analysis (Table S2). Self-interactions are denoted by arcs.

The core components of the T3S apparatus are defined by 10 operons dispersed among five different loci in the chromosome [16] (Fig. 1A). In addition, two unlinked operons encode proteins of the T3S-related flagellar export machinery. Based on protein-protein interactions defined among homologous components (Table 1) in other bacterial pathogens, the chlamydial injectisome has been proposed to have an architecture as outlined in Fig. 1B [5],[6].

To identify additional injectisome components, regulators, secretion chaperones and their respective cargo, we screened chlamydial proteins for their ability to interact with core components of the injectisome by Y2H analysis. We amplified 208 *Chlamydia*-specific, conserved hypothetical ORFs, and genes encoding putative effectors and injectisome components by PCR and cloned them into Y2H vectors to generate carboxyl terminal fusions to the DNA-binding domain (DBD) or Activator Domain (AD) of the yeast transcription factor Gal4, respectively (Table S1). For proteins containing large hydrophobic regions which tend to be toxic when expressed in yeast (e.g. inclusion membrane proteins) [28], only the putative cytoplasmic regions were expressed. MATα and MATa yeast strains expressing chlamydial proteins fused to Gal4_AD_ or Gal4_DBD_ were crossed to generate diploid strains. Positive interactions among these fusion proteins were identified by their ability to transcribe Gal4-dependent reporter genes (*GAL1_UAS_-GAL1_TATA_-HIS3*, *GAL2_UAS_-GAL2_TATA_-ADE2*) and restore growth in histidine and adenine deficient media. We identified 49 proteins that displayed various levels of homotypic and heterotypic interactions (Table S2). Most of these interactions were among proteins predicted to reside at the inner membrane and the cytoplasmic side of the basal T3S apparatus, although homotypic and heterotypic interactions were also observed among the predicted cytoplasmic domains of Inc proteins, including IncA, Ct565, Ct229 and Ct223.

### Identification of T3S chaperone binding partners

We predicted that secretory cargo proteins engaged by known T3S chaperones could be identified based on protein interactions revealed by Y2H analysis. There are three classes of T3S chaperones: class III chaperones prevent the premature polymerization of needle components in the bacterial cytoplasm [23],[29]. An interaction between the needle component CdsF and its chaperone CdsG [22] was detected in our Y2H analysis as well as an interaction between CdsG and its co-chaperone CdsE (Fig. 1C). Class II chaperones, which in *Chlamydia* include and Ct274 (LcrH/SycD-like) and the tetratricopeptide repeat (TPR) containing Scc2 and Scc3 [24], bind to hydrophobic translocator proteins [30]. We determined that Scc2 binds to both CopB and CopD while Scc3 binds to CopN (Fig. 1B). The interactions between Scc2/CopB and Scc3/CopN are in agreement with previous findings [24],[31]. In addition, we detected heterotypic interactions between Ct274 and a small (24 kDa), acidic (pI 4.5) protein Ct668. Ct274 also interacted with Ct161, a homologue of the secreted protein Lda2 [32] (Fig. 1C).

Class I chaperones are small (∼15 kDa), acidic (pI<5) proteins that assemble mostly as homodimers to bind effector proteins [25]. These chaperones have been further subclassified depending on whether they associate with one (class Ia) or several (class Ib) effectors [33]. By amino acid sequence analysis, *C. trachomatis* encodes at least three putative T3S chaperones: Ct043, Ssc1 and Ct663 (SycE/CesT-like). Unlike their counterparts in other pathogenic bacteria, we did not detect many homotypic interactions among chlamydial class I T3S chaperones. Instead, we found evidence of potential heterodimeric complexes, including an Scc1-Ct663 interaction.

Overall, these results suggest that chlamydial homologues of Class 1 T3S chaperones may form heterodimeric complexes and thus limit the usefulness of a binary Y2H screen to identify binding partners. However, given that ∼10% of the chlamydial coding potential may be devoted to T3S substrates,[12],[34],[35],[36] it is apparent that the number of effector proteins is in excess of potential chaperones. This indicates that the secretion of many chlamydial T3S substrates is either chaperone-independent or that many secretion chaperones remain to be identified.

### The C-ring component CdsQ is a central hub of multiple protein-protein interactions

CdsQ (Ct672), a homologue to the C-ring component FliN of the *Salmonella* flagellar apparatus [37] and the *Shigella* Spa33 protein [38], represented a central node of *Chlamydia* protein-protein interactions (Fig. 1D). CdsQ interacted with CdsL, an interaction that is conserved among related components in *Yersinia*, *Shigella*, and enteropathogenic *E. coli* (EPEC) injectisomes [20],[38],[39]. CdsS and CdsT were also observed to interact with CdsQ (Fig. 1D). These proteins are predicted to reside at the inner membrane and likely interact as components of the inner membrane spanning ring in other bacterial pathogens [40],[41]. An interaction between CdsQ, CdsL and CdsD has been recently reported in *C. pneumoniae* [19]. Unfortunately, we were unable to detect CdsD interacting proteins because this protein was self-activating in our Y2H reporter system. Additional components of the basal T3S apparatus may include the CdsQ-interacting proteins Ct560 and Ct567, an ORF immediately adjacent to this operon (Fig 1A). CdsQ did not interact with CdsV (Ct090), a conserved injectisome inner membrane protein, but did interact with the CdsV/FlhA homologue Ct060 (Fig. 1D). This raises the possibility that the *Chlamydia* injectisomes may be formed by a mixture of flagellar and T3S components. A combinatorial assembly of injectisomes may enhance the functionality of the secretion apparatus by adding assembly control checkpoints or by expanding the repertoire of cargo proteins that can be secreted.

We did not detect several of the predicted interactions among integral membrane components of the injectisome or the secretin CdsC. The inability to detect such interactions is likely due to the limitations of the classical Y2H system in identifying interactions among integral membrane proteins and proteins destined to fold outside of cytoplasmic compartments [42]. Nonetheless, we detected interactions between the needle component CdsF and its chaperones, CdsG and CdsE [22], and Ct584, a conserved chlamydial ORF (Fig. 1C). We postulate that these interactions were detected because they most likely occur in the bacterial cytoplasm.

Despite the known limitations of classical Y2H analysis, our ability to identify previously reported interactions validate the utility of this approach. Importantly, novel interactions identified by Y2H suggest that CdsQ may play a central role in organizing networks of proteins at the base of the injectisome, including secreted effectors.

### Ct260 and its interacting partners localize to distinct subcellular sites

T3S effectors require secretion chaperones for efficient translocation by injectisomes [43],[44]. ATPases in *Yersinia* and *Salmonella*, YscN and InvC respectively, recognize chaperone-effector complexes and provide the energy for their dissociation, thus facilitating effector protein export [45],[46]. Similarly, the inner membrane component YscU in *Yersinia* recognizes translocators as T3S export substrates [47]. Indeed, it makes intuitive sense that components at the base of the injectisome provide a platform for the recognition of secretory cargo.

Based on these observations, two chlamydial protein-protein interaction hubs linking known secreted effector proteins to CdsQ were of particular interest. In the first interaction node, Ct700, via its TPR repeat domain could act as a scaffold to dock effectors directly (e.g. Ct223 and Ct226) [34],[35] or indirectly (IncA and Lda2) [32],[48]. These protein interaction networks are linked to CdsQ via the putative protease, Ct824 (Fig. 1D). The relevance of these interactions *in vivo* remains to be determined. The other protein-protein interaction hub has Ct260 at its center and links CdsQ to the inclusion membrane proteins Cap1 [49], Ct618 [28] and Ct225 [35] (Fig. 1D). Cap1 has been postulated to be a secretion target of the chlamydial injectisome because fusion of the first 15 amino acids of Cap1 to adenylate cyclase is sufficient to impart T3S-dependent export of the fusion protein in *Shigella* [12].

**Figure 2:**
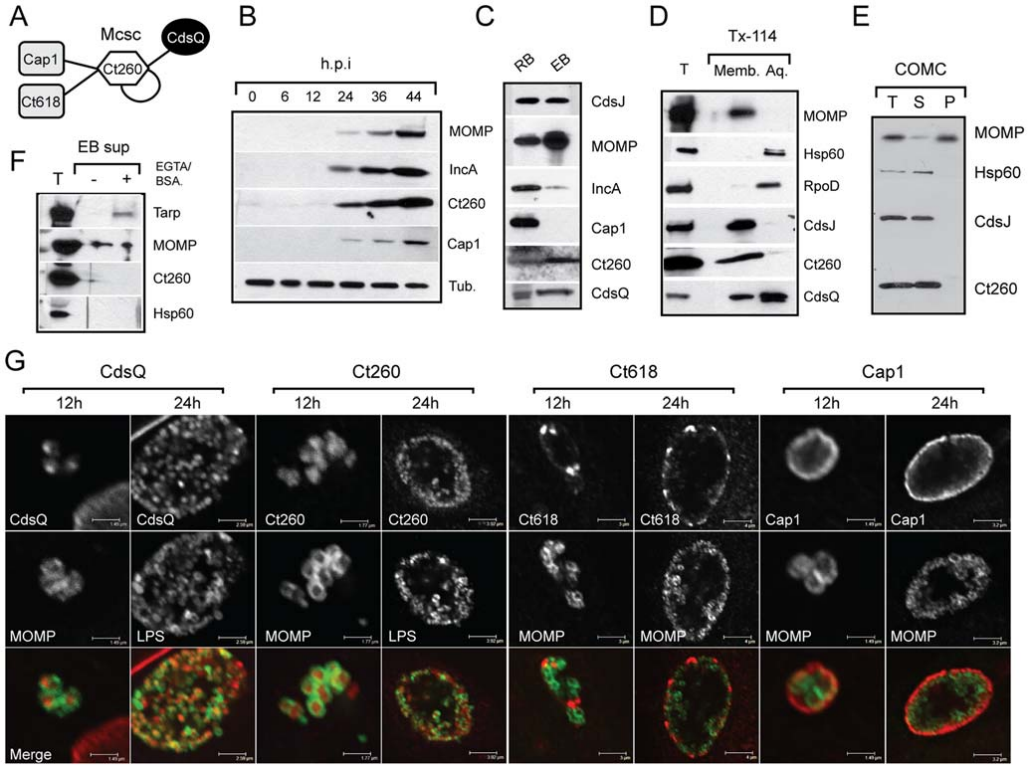
Components of a *Chlamydia* injectisome protein-protein interaction hub localize to distinct subcellular sites. (A). *Network of predicted Ct260 (Mcsc) protein-protein interactions*. Interactions were defined by Y2H analysis as described in Fig. 1D. (B–C). *Ct260 and CdsQ are expressed throughout infection*. (B). HeLa cells were infected with *C. trachomatis* and total protein samples collected at various times post-infection. Ct260, the bacterial major outer membrane protein (MOMP), and the inclusion membrane proteins IncA and Cap1 were detected with specific antibodies. Tubulin levels were serve as protein loading controls. (C). Reticulate bodies (RBs) and EBs were purified from infected cells by density gradient and total proteins were analyzed by immunoblot analysis with antibodies against the indicated chlamydial proteins. (D–E). *Ct260 and CdsQ partition with membranes and hydrophobic proteins*. (D). EBs were extracted with Triton-X114 to separate membrane from non-membrane associated proteins. Ct260 partitioned with the detergent fraction along with integral membrane proteins CdsJ and MOMP, but not the cytoplasmic proteins Hsp60 and RpoD. CdsQ partitioned in both membrane and aqueous fractions. (E). Ct260 is sensitive to Sarcosyl extraction, a detergent that solubilizes all membrane proteins except COMC, suggesting a lack of association with chlamydia outer membrane complexes (COMC) (F–G). *Ct260 is not a target of T3S*. (F). Secretion of T3S was induced by treatment of EBs with 0.5% BSA and 10mM EGTA. Tarp, but not Ct260, was detected in the extracellular media. (G). CdsQ and Ct260 localize to bacteria while Cap1 and Ct618 localize to the inclusion membrane. Ct260 and CdsQ (red) were observed exclusively in association with MOMP or LPS-positive (green) bacteria at early (12 h) and late (24 h) post-infection. Ct618 and Cap1 (red) localize to the extra-bacterial structures, including inclusion membranes. Scale bar range: 1.5 µm (12 h) to 4 µm (24 h). Abbreviations: (h.p.i.) hours post infection, (T) total lysates from purified EBs, (Memb.) and (Aq.) represent the detergent and aqueous phase of Triton-X114 extraction, (S) and (P) denote the soluble and insoluble protein fractions extracted with Sarcosyl respectively. Mcsc: Functional nomenclature for Ct260 (See Fig. 3).

To validate the novel interactions identified by Y2H, we decided to characterize the putative bindings of Ct260 to CdsQ and the secreted effectors Ct618 and Cap1 (Fig. 2A). We generated specific antisera to Ct260, and CdsQ, and determined by immunoblot analysis that these proteins are expressed in infected cells and in density gradient purified EBs and RBs (Fig. 2B&C). Next, we determined the subcellular localization of Ct260 and CdsQ in EBs by assessing their fractionation properties upon extraction from EBs with Triton-X114 or Sarcosyl [50],[51]. CdsQ and Ct260 phase partitioned with Triton-X114 indicating an association with hydrophobic proteins (Fig. 2D), although a significant portion of CdsQ was also present in the aqueous, non-hydrophobic phase. Any association of Ct260 with membranes is unlikely to include chlamydial outer membrane complexes (COMC) since the protein was readily extracted from EBs with Sarcosyl (Fig. 2E). Given its association with CdsQ and translocated effectors, Ct260 could be a core T3S component or a secreted effector. We tested the ability of Ct260 to be secreted from EBs by adapting a recently described *in vitro* secretion system. In this system, EB effectors are released into the culture supernatant by treatment with calcium chelators and bovine serum albumin at 37°C [52]. Under these conditions Ct260 was not released into the extracellular media, while Tarp, a known secreted effector, was efficiently secreted (Fig. 2F), suggesting that at least in EBs, Ct260 is not an efficient target of T3S.

We performed a detailed analysis of Ct260, CdsQ, Cap1 and Ct618 localization at 12 and 24 h post infection by immunofluorescence microscopy. Consistent with the fractionation experiments (Fig. 2D&E), CdsQ and Ct260 associated exclusively with bacteria, while Ct618 and Cap1 were found at the inclusion membrane, even at early time points (Fig. 2G). Overall, these results indicated that at steady state, Ct260 and CdsQ reside within the confines of bacterial cells, while Ct618 and Cap1 are largely exported. Therefore, any interaction between Ct260 and C618 or Cap1 is likely to be transitory and occur in the bacterial cytoplasm or in association with bacterial cytoplasmic membranes.

### Ct260/Mcsc (Multiple cargo secretion chaperone) stabilizes Ct618 and Cap1

PSI-BLAST-based database searches of Ct260 did not reveal homology to any known proteins. However, given that Ct260 is a small (18.8 kDa), acidic (pI 4.6) protein that interacted with itself and T3S effectors by Y2H analysis (Fig. 2A), we hypothesized that it was a secretion chaperone. In support of this, the predicted secondary structure of Ct260 has a similar arrangement of α-helices and β-strands as T3S chaperones (Fig. S1). We propose that Ct260 is a new T3S secretion chaperone, and thus refer to it as Multiple cargo secretion chaperone (Mcsc).

**Figure 3:**
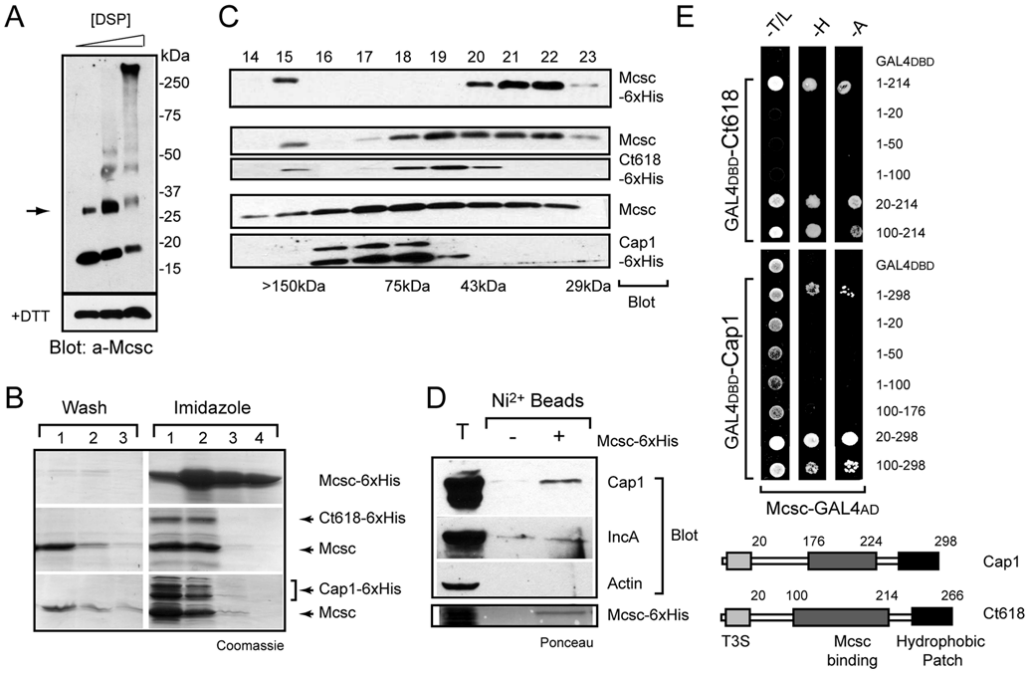
Ct260/Mcsc (Multiple cargo secretion chaperone) dimers bind to the inclusion membrane proteins Cap1 and Ct618. (A). *Mcsc forms a dimer*. Hexahistidine-tagged –Mcsc (Ct260) was treated with various concentrations of the reversible crosslinker DSP, resolved on a 4–20% gradient gel and probed with Mcsc specific antibodies. Arrow marks the dimeric form of Mcsc, which was reduced to monomeric form after boiling in the presence of DTT. (B–C). *Mcsc-Cap1 and Mcsc-Ct618 co-purify as complexes*. (B). Lysates of *E. coli* expressing 6xHis-tagged Mcsc or co-expressing Mcsc and 6xHis-tagged Ct618 (aa1–189) or Cap1 (aa1–298) were incubated with Ni^2+^-NTA agarose beads. Bound proteins were eluted with 150 mM imidazole and detected by SDS PAGE and Coomassie staining. Mcsc co-purified with Ct618-6xHis and Cap1-6xHis on Ni^2+^ beads. The numbers above the lanes represent fractions collected after incubation with wash buffer or elution buffers. Untagged Mcsc does not bind to the nickel columns non-specifically (Fig. 4E). (C). Proteins eluted from affinity columns were further fractionated by gel filtration chromatography. Mcsc eluted from the column at a size of ∼34 kDa. Mcsc-Cap1 and Mcsc/Ct618 eluted as complexes of ∼66 kDa and 54 kDa, respectively. The identity of all eluted proteins was confirmed by immunoblot analysis with anti Mcsc, Cap1 and hexahistidine tags. (D). *Mcsc binds to endogenous Cap1 from infected cells*. Lysate of infected HeLa cells were incubated with purified Mcsc dimers pre-bound to Ni^2+^-NTA agarose beads, and proteins were eluted with 150 mM imidazole. Cap1, but not IncA, preferentially co-eluted with Mcsc. Ponceau staining of nitrocellulose membrane indicate levels of Mcsc eluted from beads. (E). *The central region of Ct618 and Cap1 mediate binding to Mcsc*. The Mcsc binding domains of Ct618 and Cap1 were mapped by Y2H analysis. Positive interactions were assessed by activation of GAL4-dependent *HIS3* and *ADE2* reporter genes and growth in media lacking histidine (H) or adenine (A). Growth on media lacking tryptophan (T) and leucine (L) are shown as controls for maintenance of the Y2H vectors. Cartoon schematic shows Mcsc binding region maps adjacent to the large hydrophobic region at the COOH-terminus of these inclusion membrane proteins.

We first tested if recombinant Mcsc formed homotypic complexes by treating recombinant protein with low levels of the chemical crosslinker DSP on ice and assessing the formation of higher molecular weight complexes by SDS PAGE and immunoblots. Mcsc was readily cross-linked into a major 36 kDa molecular weight species consistent with the formation of a dimer (Fig. 3A). This dimeric form of Mcsc was seen even in the absence of chemical crosslinkers (Fig. 3A) and its relative abundance was sensitive to heat and reducing agents (not shown). Higher order complexes consisting of potential trimers and tetramers were also observed at the highest concentration of crosslinker. The relevance of these higher order complexes is not clear since we have observed a cold-induced aggregation of purified Mcsc (not shown). Next, we determined if Mcsc associated with the effectors Cap1 and Ct618 by co-expressing untagged Mcsc and a hexahistidine-tagged versions of full-length Cap1 (aa 1–298) and the amino-terminal domain of Ct618 (aa1–189) from bicistronic vectors. Mcsc efficiently co-purified with 6xHis-tagged Ct618 or Cap1 on Ni^2+^ NTA resin, suggesting a stable interaction between these inclusion membrane proteins and the putative chaperone (Fig. 3B). We sized these complexes by gel filtration chromatography and determined that when expressed alone, Mcsc eluted as a ∼35 kDa complex, a size consistent with that of the dimeric forms identified in cross-linking experiments (Fig. 3C). In contrast, when Mcsc was co-expressed with Cap1 or Ct618, it fractionated as a protein complex of 66–78 kDa and 54–65 kDa respectively (Fig. 3C). The size of these complexes is consistent with that of two Mcsc subunits bound to one effector protein.

Next, we tested if Mcsc could bind to full-length Cap1 from infected cell lysates. We incubated NP40 solubilized membranes from infected cells with recombinant 6xHis-tagged Mcsc dimers, and assessed the ability of endogenous Cap1 to be purified on Ni^2+^NTA agarose. Cap1, but not the inclusion membrane proteins IncA, specifically bound to Ni^2+^-beads in the presence of Mcsc (Fig. 3D).

We mapped the regions of Ct618 and Cap1 that interact with Mcsc by Y2H analysis. Truncated forms of Ct618 and Cap1 were fused to the GAL4_DBD_ and co-expressed in yeast with GAL4_AD_-Mcsc. Positive interactions were assessed by growth in histidine or adenine deficient media as described above. A region encompassing the central region of both Ct618 and Cap1 was sufficient to mediate an interaction with Mcsc (Fig. 3E). The Mcsc binding site on its secretory cargo (Ct618 and Cap1) is within the range of what has been observed in other chaperone-effector protein complexes [44].

**Figure 4:**
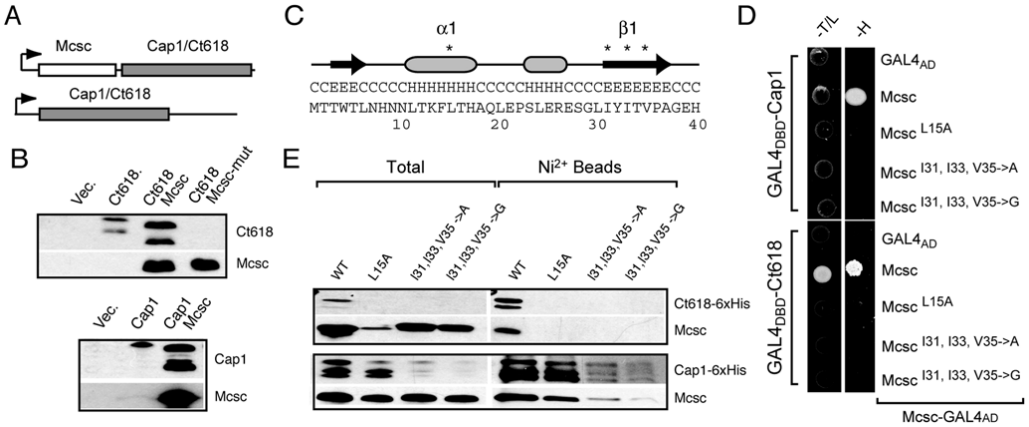
Effector protein stability is linked to Mcsc function. (A). *Schematic of T7 expression vectors used to test the effect of Mcsc in Ct618 and Cap1 expression* (B). *Ct618 and Cap1 display altered mobility in the presence of Mcsc*. Ct618 and Cap1 were expressed with or without Mcsc and total proteins (∼30 µg) were analyzed by immunoblots with specific antibodies. Note decreased levels of expression and altered sizes of Cap1 and Ct618 in the absence of Mcsc. (C–E) *Mcsc mutations in the putative effector binding domain disrupt cargo binding and destabilize Cap1 and Ct618*. (C). Point mutations in the putative effector binding regions of class I chaperones [44] were generated in the α1 (L15A) and β1 strand (L31, L33 & L35 to A or G) either in Y2H (D) or in *E. coli* expression vectors (E). (D). All Mcsc mutants disrupted the Y2H interactions between Mcsc and Cap1 and Ct618. (E). *E. coli* co-expressing 6xHis-tagged Ct618 or Cap1 with Mcsc mutants were monitored for steady-state expression of recombinant proteins and co-purification on Ni^2+^ agarose beads. Mutations in the Mcsc β1 strand led to substantially lower steady-state levels of Ct618 and Cap1. In contrast, L15A mutants altered Ct618, but not Cap1, stability.

Our initial attempts at purifying recombinant Cap1 and Ct618 indicated that these proteins were not stable when expressed at high levels in *E.coli*. Given our finding that Mcsc may constitute a chaperone for these effectors, we compared the effect of expressing Cap1 and Ct618 with or without Mcsc from mono and bicistronic *E. coli* expression vectors under the control of a T7 promoter (Fig. 4A). Consistent with our earlier observations, the detected levels of Cap1 and Ct618 protein were significantly lower in the absence of Mcsc (Fig. 4B). Interestingly, Cap1 and Ct618 migrated at a higher apparent molecular weight in the absence of Mcsc (Fig. 4B), suggesting that in the presence of the chaperone, these inclusion membrane proteins may be subjected to conformational changes that expose susceptible regions to *E. coli* proteases. Although this processing may be specific to *E. coli*, it is clear that the expression and stability of Cap1 and 618 is dependent on Mcsc, thus establishing its role as a chaperone.

Based on the crystal structures of various class I secretion chaperones, conserved hydrophobic amino acids in the first α-helix and β-strand are proposed to be important for the recognition of secretory cargo [44]. We identified the corresponding amino acids in Mcsc (Fig. 4C & S1) and tested if these residues played a role in the binding of Mcsc to Ct618 and Cap1. First, we generated point mutation in Mcsc's α1-helix (L15A) and β1-strand (I31A, I33A V35A (“3A”) and I31G, I33G V35G (“3G”)) and tested the ability of these mutants to interact with effectors by Y2H. Consistent with their proposed role in binding substrates, all three mutations in Mcsc significantly impaired interactions with Cap1 and Ct618 (Fig. 4D). Next, we introduced these mutations into Mcsc-Cap1 and Mcsc-Ct618 bi-cistronic *E. coli* expression vectors (Fig. 4A). The 3A and 3G mutations did not affect the expression of Mcsc (Fig. 4E) or its ability to form dimers (not shown). However, co-expression of 3A and 3G Mcsc mutants led to significantly lower levels of Ct618 and Cap1 (Fig. 4E) as assessed by immunoblot analysis of total protein lysates. In contrast, co-expression with the L15A Mcsc mutant had little effect on the total amounts of Cap1observed but Ct618 was not detectable (Fig. 4D). We tested if these mutants were still capable of binding either hexahistidine tagged Cap1 or Ct618 by co-isolating effector complexes on Ni-NTA agarose beads. As shown in Fig. 4D, Mcsc and Mcsc mutants co-purified with Cap1 in a manner proportional to the levels of Cap1 present in the total lysates. Not surprisingly, Mcsc mutants did not co-purify with Ct618, since the effector was not detectable in the total samples. It is worth noting that Mcsc 3A and 3G mutants had a much greater impact on Cap1 and Ct618 expression levels than the absence of Mcsc, suggesting that Ct618 and Cap1 levels are directly affected by their interaction with Mcsc (Fig. 4B & E). We postulate that 3A and 3G Mcsc mutants, like the wild type counterpart, still provides a platform for the binding of Cap1 and Ct618, but the binding interaction is not strong enough to prevent their dissociation from Mcsc and eventual degradation.

### CdsQ binds to Mcsc and Mcsc-Cap1 protein complexes

**Figure 5:**
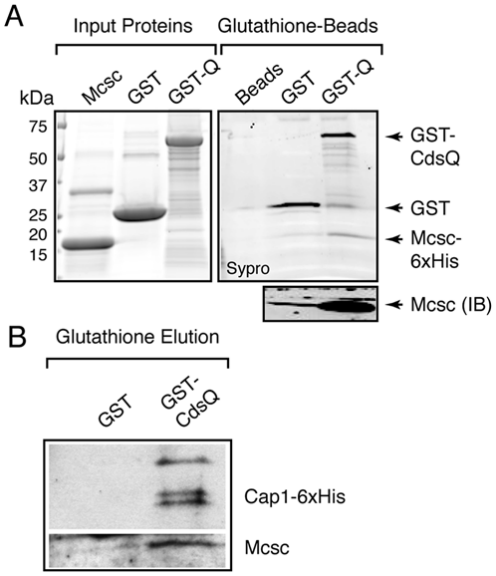
Mcsc-effector complexes bind to CdsQ, the C-ring component of the injectisome. (A). *CdsQ binds to Mcsc*. Recombinant GST or GST-CdsQ coupled to glutathione Sepharose beads was incubated with 6xHis-tagged Mcsc dimers. Bound proteins were eluted with 5 mM reduced glutathione and analyzed by SDS PAGE followed by Sypro Orange staining and immunoblots (IB). Arrowheads mark GST-CdsQ, GST and 6xHis-Mcsc. Mcsc was preferentially co-precipitated in the presence of CdsQ. (B). *CdsQ binds to Mcsc-Cap1 effector complexes*. Pull-down assays were done as in (A) but using Mcsc/Cap1-6xHis protein complexes purified by gel filtration chromatography (Fig. 3C). By immunoblot analysis, Mcsc/Cap1 protein complex co-purified only in the presence of GST-CdsQ.

T3S chaperones are proposed to play a role in targeting secretory cargo to the injectisome, either by providing new targeting information [53], or facilitating the exposure of the short amino terminal export signal [6]. Based on these findings we were intrigued by the significance of Mcsc-CdsQ interactions identified by Y2H. First, we confirmed these interactions by demonstrating that purified Mcsc dimers efficiently bound to GST-CdsQ, indicating that the presence of the effector is not required for Mcsc to engage the C-ring (Fig. 5A). Next, we tested if CdsQ can bind to Mcsc-effector complexes by incubating GST-CdsQ with Mcsc-Cap1 complexes isolated by gel filtration chromatography. A complex of Mcsc and Cap1 co-eluted from glutathione sepharose beads when incubated with GST-CdsQ but not GST alone (Fig. 5B). Based on CdsQ's homology to the C-ring component Spa33, which localizes to the base of *Shigella* injectisome [38], we postulate that CdsQ associates with the base of the chlamydial secretion needle apparatus and provides a platform for the recognition of Mcsc and Mcsc-effector protein complexes.

## Discussion

Greater than 10% of the *Chlamydia* genome is predicted to encode for substrates of T3S [14],[54]. This arsenal of effectors is required for efficient cell invasion, establishment of the inclusion, acquisition of nutrients, and avoidance of innate immune responses. How these highly adapted pathogens coordinate the secretion of effector proteins is largely unknown. In one model, a hierarchy of effector secretion is established by the timing of their synthesis. In this manner, temporal gene expression over the early, mid and late cycles dictates effector delivery [14],[55]. One caveat with this model is that early cycle genes continue to be expressed throughout infection [55]; therefore, all effectors are theoretically present by mid-late cycle (∼20 h for urogenital *C. trachomatis* serovars). The presence of early, mid and late effectors competing to engage a common injectisome would argue that additional components are involved to ensure an orderly translocation of effectors. How do mid-late effectors compete for efficient transport given that all these effectors must engage a common injectisome?

Most effector proteins contain a T3S targeting signal at their extreme amino terminus that is broadly recognized by divergent injectisomes. Additional targeting information contained within approximately the first 200 amino acid residues of the effector provides binding sites for secretion chaperones [44],[56],[57]. These chaperones are multi-functional: they target effectors to the proper injectisome, stabilize pre-formed effector proteins, mask membrane targeting domains prone to aggregation and possibly impart a translocation hierarchy [43],[58],[59]. T3S chaperones are small, acidic proteins that display a conserved α−β−α fold and form stable homo- and heterodimers [60]. These dimers bind to partially unfolded effector proteins via a hydrophobic patch formed by residues on the α1, β1 and β4-5 strands [44].

Despite the limited homology among T3S chaperones, six potential *C. trachomatis* chaperones can be identified based on primary amino acid sequences: Ct043, Scc1 (Ct088), Scc2 (Ct568), Scc3 (Ct860), Ct274 and Ct663 [61]. To identify their potential effector cargo, we screened for interacting chlamydial proteins by Y2H analysis. We confirmed previously reported interactions between Scc2 and CopB, and Scc3 and CopN [24],[31] (Fig. 1C). In addition, we identified a novel interaction between Scc2 and CopD and determined that Ct274 may interact with two small acidic proteins, Ct161 and Ct668 (Fig. 1C). Ct668, encoded within an operon of structural injectisome components, was previously identified in a screen for proteins exported from the inclusion [28]. PSI-BLAST analysis indicates that Ct668 is related to a large family of hypothetical DNA-binding proteins, raising the intriguing possibility that Ct668 is a transcriptional regulator. Whether Ct668 is a chaperone, a core T3S component, or a regulatory factor remains to be determined.

Given the large number of effectors and the dearth of T3S chaperone interacting partners identified by bioinformatics, we hypothesized that many secretion accessory factors remained to be found. We speculated that these factors may act as adaptors between effector proteins and components of the injectisome. In a subgenomic interactome map of the T3S apparatus, CdsQ emerged as a central node of protein-protein interactions (Fig. 1D). Based on its homology to FliN and Spa33, CdsQ is predicted to form the C-ring, a large protein scaffold at the base of the injectisomes. Interestingly, many of the CdsQ interacting factors we identified are conserved among injectisomes [18], suggesting small deviations in the basic architecture of this secretion apparatus despite the large evolutionary distance between Chlamydiales and other Gram-negative bacteria [17]. We identified two novel hubs of protein-protein interactions (Ct260 and Ct700) that linked multiple inclusion membrane proteins to the secretion apparatus (Fig. 1D). Ct260/Mcsc was of particular interest because, in addition to its direct interaction with CdsQ and many secretory cargo proteins (Cap1, Ct225 and Ct618) [35],[49],[54], it had predicted secondary structural similarity to other T3S chaperones (Fig. S1). Mcsc is expressed in both developmental forms of *Chlamydia* (Fig. 2C) and forms stable dimers and complexes with at least two inclusion membrane proteins (Fig. 3A–D). In *E. coli*, significantly lower levels of Ct618 and Cap1 were observed in the absence of Mcsc or when Mcsc was mutated to impair effector protein binding (Fig. 4B, D, E). These findings suggest that one of the functions of Mcsc is to stabilize these inclusion membrane proteins. While the data presented here implies that Mcsc acts as a bona fide Class I T3S chaperone, at present we cannot assign a role for Mcsc in directing the secretion of Cap1 or Ct618. Proof of such a role will require the development of a proper system for genetic manipulation in *Chlamydia* or the reconstitution of chaperone-dependent secretion in a heterologous T3S system.

**Figure 6:**
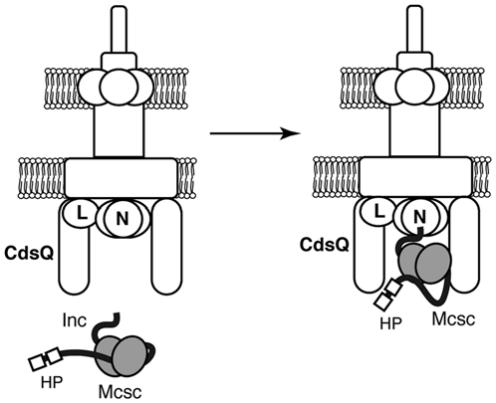
Model of Mcsc recognition by the *Chlamydia* injectisome. The inclusion membrane proteins Cap1 and Ct618 in complex with Mcsc dimers engage the C-ring of the injectisome (CdsQ). These docked complexes are presumably placed in proximity to the ATPase CdsN to initiate secretion of the effector proteins. HP: Hydrophobic patches common to many inclusion membrane proteins (Inc).

In *S. typhimurium*, the SptP-SicP effector chaperone complex binds to the conserved ATPase InvC [45]. InvC then dissociates this complex and provides the energy required for the translocation of the SptP across the injectisome. A similar role for the ATPase in substrate selection by chaperone binding has been proposed for the EPEC T3S system and flagellar export [62]. By analogy to these findings, we propose that CdsQ recruits Mcsc-effector complexes directly to the base of the injectisome (Fig. 6). However, because Mcsc can bind to the C-ring component CdsQ in the absence of its bound substrate (Fig. 5A), it is also possible that Mcsc is pre-docked on the C-ring of the injectisome. This latter possibility is consistent with the observation that Mcsc partitions with inner membranes in EBs (Fig. 2D) in the absence of its cognate cargo, which are not synthesized until after 1 h (Cap1) and 8 h (Ct618) post invasion [55]. Because the stability of effectors require binding by Mcsc, either Mcsc dimers detach from the injectisome and bind to newly synthesized effectors at distal sites or the translating ribosome itself is recruited to the C-ring. In support of a localized translation model, the chlamydial GTPase (HflX) that binds to the 50S ribosome subunit, localizes to the bacterial inner membrane [63], suggesting that some ribosomes may be pre-docked at secretion sites. Furthermore, one of the CdsQ-interacting proteins identified by Y2H is Ct677, a putative ribosome recycling factor (Fig. 1D). These findings raise the intriguing possibility that the injectisome may interact with the bacterial translational machinery. As an obligate intracellular pathogen, it would not be surprising if the chlamydial injectisome, the main portal for communication with the host, is hard wired to interface with the bacterial and transcriptional and translational machinery. Such a system would allow for rapid regulation of effector protein translocation in response to intracellular (pathogen's metabolic status, developmental cues) and extracellular (host responses, inclusion lumen environment) signals.

The prominence of CdsQ as a hub of protein-protein interactions, including secretion chaperones, suggests a central role in regulating the recognition of effector proteins. In the flagellar system, the CdsQ-related C-ring component FliM binds to the flagellar general chaperone FliJ [64]. Similarly, the *Shigella* CdsQ homologue, Spa33, can be co-isolated with effector proteins indicating that recognition of chaperone-effector complexes by C-ring components may be evolutionarily conserved [38]. Based on these observations and our own findings with the *Chlamydia* injectisome, we hypothesize that proteins at the base of the translocon integrate multiple intracellular signals to regulate the production and secretion of virulence factors. Interestingly, in the flagellar systems, the C-ring protein FliG was recently shown to bind to fumarate reductase to control the direction of flagellar rotation in response to fumarate [65]. These findings suggest that in addition to the recognition of secretion substrates, the C-ring may provide a mechanism for the integration of intracellular cues by binding to “sensor” proteins not typically thought to be associated with T3S (e.g. envelope assembly, intermediary metabolism, stress). Global screens for proteins that interact with components at the base of the injectisome, such as those described in this study, should help elucidate the intricacies of T3S function and regulation.

## Materials and Methods

### Bacterial strains and plasmids

Bacteria, yeast strains and plasmids are listed in Supplementary Table S3. *C. trachomatis* ORFs were amplified from purified *C. trachomatis* serovar D genomic DNA using the primers listed in Table S3 and the Expand High Fidelity PCR system (Roche). PCR products were subcloned into pET24d, pET15b (Novagen) and/or pGEX-4T1 (Invitrogen). Mutation sites, L15→A, 3A (I31, I33, V35→A) and 3G (I31, I33, V35→G), were chosen based on sequence alignments of several T3S chaperones (Fig. 4C & S1) and site directed mutagenesis was performed with QuickChange Kit (Stratagene) as instructed by the manufacturer. Mcsc/Cap1-6xHis, Mcsc/Ct618-6xHis and Mcsc(L15A, 3A or 3G)/Ct618-6xHis were vo-expressed as a bicistronic construct in pET-24d(+). Affinity-tagged recombinant proteins were expressed in *E. coli* BL21 DE3 (Stratagene) with 0.5 mM IPTG.

### Creation of Y2H strains library and Y2H screens

The Matchmaker Two-Hybrid System (Clontech) was used in this screen. *C. trachomatis* genes (CT) were amplified from a previously established chlamydial ORF library [28] using the pGAD and pGBT9 primers or directly from genomic DNA using ORF specific primers (Table S3) with Expand High Fidelity polymerase. PCR products were transformed into both Y2H reporter yeast strains PJ69-4a (MATα) or AH109 (MATa) along with digested Y2H vectors pGAD424 or pGBT9 by the lithium acetate method [66]. MATα yeast strains containing *C. trachomatis* ORFs inserted into pGAD424 were arrayed in 96 well plates. This ordered array of yeast strains was mated against individual MATa yeast strains containing pGBT9-CT ORFs. The resulting diploids were selected in synthetic complete (SC) medium lacking leucine and tryptophan. Yeast matings were performed semi-automatically with a RoboMEK-FX (Beckman-Coulter) liquid handler. Positive interactions were assessed by monitoring growth on SC media lacking histidine (His) or adenine (Ade) over 4 days.

### Cell culture conditions and propagation of *C. trachomatis*

HeLa cell monolayers were grown in Dulbecco's minimal essential medium (DMEM)(Gibco) supplemented with 10% fetal bovine serum (Mediatech) at 37°C/5% CO_2_. *C. trachomatis* LGV (serotype L2) EBs were purified by density gradient centrifugation as previously described [51]. Infections were initiated by adding EBs to HeLa monolayers (multiplicity of infection (MOI) ∼1) followed by centrifugation at 3,500×*g* for 25 min at 10°C.

### Generation of antibodies and immunodetection methods

GST-Mcsc, GST-CdsQ and GST-Ct618 (aa1–96) were expressed in *E. coli* and purified with glutathione coated Sepharose 4-Fast Flow beads (GE Healthcare) at 4°C for 2 hr. Recombinant GST-Mcsc was eluted with 20 mM reduced glutathione in PBS (pH 8.0) and used to immunize female White New Zealand rabbits (5∼6 lbs) (Robinson Services, Inc.). Antisera was depleted of anti-GST antibodies and affinity purified over a GST-Mcsc or GTS-CdsQ column. Bound antibodies were eluted with 0.2 M Glycine (pH 2.5) and neutralized with 1 M K_2_HPO_4_. The resulting antibodies was then dialyzed in PBS and stored in −80°C. For immunoblot analysis, protein samples were separated by SDS-PAGE, transferred to 0.45 µm nitrocellulose membranes and blocked in 2% non-fat powder milk in TBST (50 mM Tris-Base, 150 mM NaCl, 0.2% Tween pH 7.4). Membranes were incubated with primary antibody diluted in 1% milk-TBST, followed by incubation with secondary antibody conjugated to horseradish peroxidase and detection by chemiluminescence (Pierce). Primary antibodies include anti-Tarp (T. Hackstadt, RML), Hsp60, MOMP and CdsJ (K. Fields, U. of Miami), Cap1 (A. Subtil, Pasteur Institute), RpoD (M. Tang, UC Irvine), actin (Sigma-Aldrich), α-tubulin (Sigma-Aldrich), hexahistidine (Rockland Inc.) and IncA (generated in our laboratory).

For indirect immunofluorescence detection, HeLa cells were seeded on glass coverslips and infected at MOI∼1 for the indicated times. Infected cells were fixed in methanol, blocked with 2% bovine serum albumin (BSA) in PBS and incubated with polyclonal anti-Mcsc, CdsQ, Ct618 or Cap1 antibodies (1∶100 in PBS/2% BSA) and mouse monoclonal anti-MOMP antibodies (RDI) (1∶300 in PBS/2% BSA) for 1 hr at 4°C. Immunoreactive material was detected with Alexafluor conjugated secondary antibodies (Invitrogen, CA). Images were acquired with a Leica TCS SL confocal microscope and processed with Leica imaging software.

### Subcellular fractionations

#### Outer membrane complexes

Density gradient purified EBs were solubilized in 2 mls of Sarcosyl extraction buffer (PBS pH 7.4, 2% Sarcosyl, 1.5 mM EDTA) at 37°C for 1 h. The resulting lysates were centrifuged at 100,000×*g* for 1 h at 4°C. Sarcosyl-insoluble pellets (*Chlamydia* Outer Membrane complexes-COMC [51]) were washed three times in Sarcosyl extraction buffer. COMC pellets were resuspended in PBS pH 7.4/10 mM MgCl_2_ and incubated with 25 µg of DNase at 37°C for 2 hrs. COMC pellets were centrifuged as described above and resuspended in PBS. Sarcosyl insoluble and soluble protein fractions were acetone precipitated and resuspended in SDS-PAGE loading buffer prior to immunoblot analysis.

Triton X114 fractionation of membrane proteins. EBs (∼10^10^ IFUs) were centrifuged at 20,000×*g* for 5 min at 4°C, resuspended in 50 µl of PBS/5 mM dithiotheritol (DTT) for 10 min at 4°C, and integral membrane proteins were extracted using 1% Triton-X114 as previously described [50]. The resulting aqueous fraction containing cytoplasmic proteins and the detergent fraction containing membrane proteins were acetone precipitated and resuspended in SDS-PAGE loading buffer.

*In vitro* induction of T3S. In vitro secretion of T3S substrates was performed as described [52]. Briefly, density gradient purified EBs were washed 5 times in potassium acetate (KAc) buffer (50 mM potassium acetate, pH 4.8) and resuspended in KAc with or without 0.5% BSA/10 mM EGTA. After 30 min incubation at 37°C, bacteria were centrifuged at 20,000×*g* for 5 min at 4°C. Supernatants were re-centrifuged and the secreted proteins in the clarified samples were subjected to immunoblot analysis.

### Biochemical analysis of Mcsc

#### Crosslinking

Purified 6xHis-Mcsc was treated with the reversible chemical crosslinker DSP (dithiobis[succinimidylpropionate]) (Pierce) at concentrations between 0.25–1 mM in Dimethyl sulfoxide (DMSO). After 20 min incubation at 4°C, reactions were quenched in TNE and solubilized in sample buffer devoid of reducing agents. The samples were divided equally and 50 mM dithiothreitol (DTT) was added to half of the samples. Samples were analyzed by immunoblot.

#### Purification of Mcsc and Mcsc/effector protein complexes

Mcsc, Ct618-Mcsc, and Cap1-Mcsc complexes were purified from bacteria expressing pET15b-Mcsc, pET-24d-Mcsc/Cap1 or pET24d-Mcsc/CT618 by incubation of clarified supernatants with Ni^2+^-nitriloacetic acid (NTA) agarose beads (Qiagen, Ca). Beads were washed 4 times with wash buffer (50 mM Phosphate buffer pH 8.0, 150 mM NaCl, 10 mM Imidazole) and recombinant proteins were eluted with 4×0.5 ml of 50 mM Phosphate Buffer containing 150 mM NaCl and150 mM imidazole. Co-eluted proteins were identified by Coomasie staining and immunoblots with anti-Mcsc and anti-6xHis antibodies. For gel filtration, hexahistidine-tagged Mcsc, Cap1/Mcsc or CT618/Mcsc protein samples were loaded on a 16/60 Sephacryl S-100 HR column (GE Healthcare) and proteins eluted in 10 mM Tris-HCl/50 mM phosphate Buffer/150 mM NaCl pH 8.0 at a flow rate of 0.5 ml/min and at a pressure of 0.5 MPa. Proteins were collected in fraction sizes of 2.5 ml and detected with anti-6xHis or the anti-Mcsc antibodies. Size of the eluted complexes was determined with a low molecular weight gel filtration calibration kit (GE Healthcare).

#### Mcsc-Cap1 pull-down assays

HeLa cell monolayers were infected with *C. trachomatis* serovar L2 at an MOI of ∼5 for 28 hours, lysed in 1% NP40 (50 mM Tris pH 7.5, 50 mM NaCl, 1 mM PMSF, protease inhibitor cocktail). Approximately 400 µg of lysates from infected or uninfected cells were incubated with 50 µg of purified 6xHis-Mcsc at 4°C for 30 min and isolated with Ni^2+^-NTA agarose beads. The beads were washed five times with binding buffer and bound proteins were subjected to immunoblot.

### GST-CdsQ binding assays

Freshly purified GST or GST-CdsQ coupled to glutathione sepharose beads was incubated with 6xHis-Mcsc dimers or Mcsc/Cap-6xHis complexes purified by gel filtration and incubated overnight at 4°C in binding buffer (50 mM Tris-HCl, 150 mM NaCl, 2 mM EDTA and 0.5% TritonX-100) After extensive washing, bound proteins were eluted with 25 mM glutathione and subjected to SDS PAGE followed by staining with Sypro orange (Molecular Probes) and detection with a Typhoon 9410 Phosphoimager, (GE Healthcare). Parallel samples were analyzed by immunoblot.

## Supporting Information

Table S1 Summary of chlamydial genes characterized by yeast two-hybrid analysis
(0.36 MB DOC)

Table S2 Summary of Y2H-based protein-protein interactions identified between chlamydial proteins
(0.18 MB DOC)

Table S3 Strains, plasmids and primers
(0.06 MB DOC)

Figure S1 Mcsc structural models. A–C. Predicted three dimensional structure of Mcsc. A putative 3D model of Mcsc (yellow) was generated with the neural network folding prediction program I-TASSER (http://zhang.bioinformatics.ku.edu/I-TASSER/) [1]. Modeling of Mcsc and SigE structure were performed with PyMOL (http://www.pymol.org/). Overlay of Mcsc monomers (A) on SigE (magenta) monomers and dimers (blue) (B), and a model of a Mcsc dimer (C). D. Conservation among amino terminal residues in Class I T3S chaperones required for effector protein binding. Conserved amino acids (blue) in Mcsc align with α-helix and β-strand 1 as assessed by secondary structure predictions performed with PSIPRED (http://bioinf.cs.ucl.ac.uk/psipred/).
(3.34 MB TIF)
