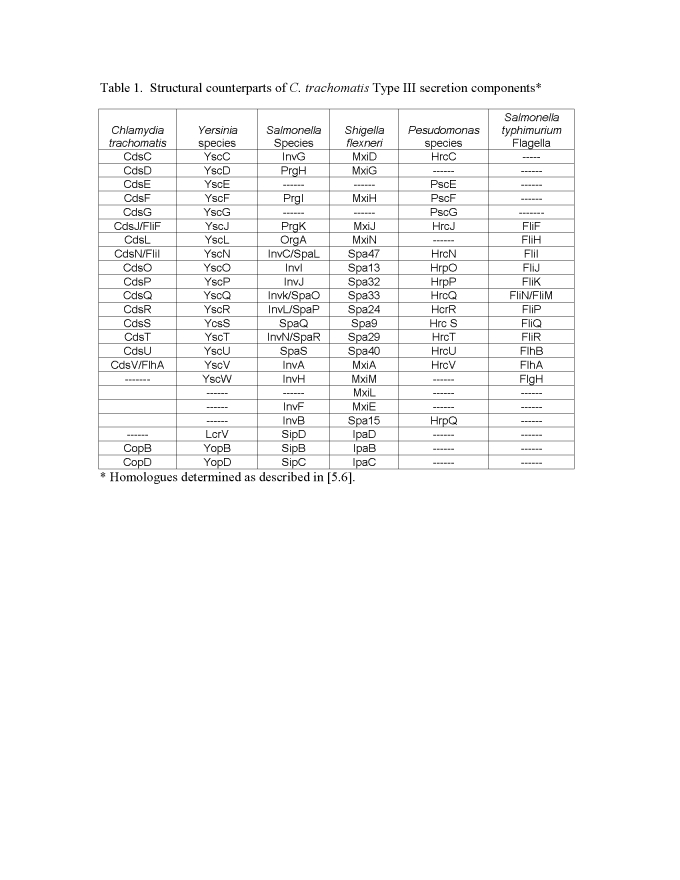# Correction: The *Chlamydia* Type III Secretion System C-ring Engages a Chaperone-Effector Protein Complex

**DOI:** 10.1371/annotation/7e073adb-7483-43be-88ec-e318399e76c2

**Published:** 2009-10-13

**Authors:** Kris E. Spaeth, Yi-Shan Chen, Raphael H. Valdivia

Table 1 was not included in the published article. The table is available here: 

**Figure:**