# Supplementary material for: The Chlamydia Type III Secretion System C-ring Engages a Chaperone-Effector Protein Complex
Source: PLoS Pathog. 2009 Sep 11;5(9):e1000579. doi: 10.1371/journal.ppat.1000579 (PMC2734247; doi:10.1371/journal.ppat.1000579)
Supplement: Table S1 — Summary of chlamydial genes characterized by yeast two-hybrid analysis (0.36 MB DOC) [file ppat.1000579.s001.doc]

Table S1: Summary of chlamydial genes characterized by yeast two-hybrid analysis

| **ORF^a^** | **ORF Length (aa)^b^** | **Region cloned (aa)^c^** | **Inserted into pGAD424^d^** | **Inserted into pGBT9^e^** |
| --- | --- | --- | --- | --- |
| CT005 | 363 | 169-363 | YES | YES |
| CT006 | 183 | 1-189 | ------- | YES |
| CT007 | 316 | 1-316 | YES | YES |
| CT036 | 403 | 80-403 | YES | YES |
| CT037 | 118 | 1-118 | YES | YES |
| CT043 | 167 | 1-167 | YES | YES |
| CT049 (Pls1) | 490 | 1-490 | YES | YES |
| CT050 (Pls2) | 536 | 1-536 | YES | YES |
| CT058 | 367 | 73-367 | YES | YES |
| CT059 (Fer) | 91 | 1-91 | YES | YES |
| CT060 (FlhA) | 605 | 1-605 | YES | YES |
| CT061(FliA) | 253 | 1-253 | YES | YES |
| CT062 (TyrS) | 412 | 1-412 | YES | YES |
| CT066 | 158 | 1-158 | YES | ------- |
| CT080 | 97 | 1-97 | YES | YES |
| CT082 | 560 | 1-560 | YES | ------- |
| CT083 | 160 | 1-160 | YES | ------- |
| CT084 | 361 | 1-361 | YES | YES |
| CT085 | 579 | 1-579 | YES | ------- |
| CT087 | 527 | 1-527 | YES | YES |
| CT088 (Scc1) | 146 | 1-146 | YES | YES |
| CT089 (CopN) | 421 | 1-421 | YES | YES |
| CT090 (CdsV) | 708 | 1-708 | YES | ------- |
| CT091 (CdsU) | 360 | 1-360 | YES | ------- |
| CT092 (YchF) | 366 | 1-366 | ------- | YES |
| CT103 | 298 | 1-298 | YES | YES |
| CT113 | 867 | 1-867 | YES | ------- |
| CT115a (IncD) | 141 | 1-40 | YES | YES |
| CT115b (IncD) | 141 | 93-141 | YES | YES |
| CT116 (IncE) | 132 | 99-132 | YES | YES |
| CT117 (IncF) | 104 | 1-23 | YES | YES |
| CT118 (IncG) | 167 | 93-167 | YES | YES |
| CT118 FL | 167 | 1-167 | YES | ------- |
| CT119 (IncA) | 273 | 109-273 | YES | YES |
| CT120 | 80 | 1-80 | YES | YES |
| CT134 | 137 | 1-137 | YES | YES |
| CT136 | 239 | 1-239 | YES | YES |
| CT144 | 285 | 1-285 | YES | YES |
| CT146 | 663 | 1-663 | YES | YES |
| CT147 | 1449 | 898-1449 | YES | YES |
| CT148 | 507 | 1-507 | YES | YES |
| CT149a | 315 | 1-185 | YES | YES |
| CT149b | 315 | 204-315 | YES | YES |
| CT155 | 313 | 1-313 | ------- | YES |
| CT156 (Lda1) | 113 | 1-113 | YES | ------- |
| CT157 | 404 | 1-404 | YES | YES |
| CT158 | 238 | 1-238 | YES | YES |
| CT159 | 310 | 1-310 | ------- | YES |
| CT160 | 167 | 1-167 | YES | YES |
| CT161 | 246 | 1-246 | YES | YES |
| CT163a (Lda3) | 548 | 83-548 | YES | YES |
| CT163 (Lda3) | 548 | 1-548 | ------- | YES |
| CT164 | 86 | 1-86 | YES | YES |
| CT165 | 148 | 1-148 | YES | ------- |
| CT168 | 100 | 1-100 | YES | YES |
| CT173 | 90 | 1-90 | YES | YES |
| CT179 | 170 | 48-170 | YES | YES |
| CT181 | 236 | 22-236 | YES | ------- |
| CT191 | 116 | 1-116 | YES | YES |
| CT192 | 257 | 107-257 | ------- | YES |
| CT194 | 470 | 1-293 | YES | ------- |
| CT195 | 363 | 212-363 | YES | YES |
| CT197 | 338 | 1-338 | YES | YES |
| CT214 | 547 | 101-547 | YES | YES |
| CT222 | 129 | 89-129 | YES | YES |
| CT223 | 270 | 86-270 | YES | YES |
| CT224 | 147 | 84-147 | YES | YES |
| CT225 | 122 | 69-122 | YES | YES |
| CT226 | 176 | 99-176 | YES | YES |
| CT227 | 133 | 87-133 | YES | YES |
| CT228 | 196 | 88-196 | YES | YES |
| CT229 | 215 | 92-215 | YES | YES |
| CT233 (IncC) | 178 | 1-97 | YES | YES |
| CT234 | 928 | 417-928 | YES | YES |
| CT235 | 137 | 1-137 | YES | ------- |
| CT244 | 398 | 1-398 | YES | YES |
| CT249 | 116 | 1-98 | YES | YES |
| CT253 | 215 | 1-215 | YES | YES |
| CT256 | 414 | 128-414 | YES | YES |
| CT257 | 404 | 114-404 | YES | YES |
| CT259 | 248 | 1-248 | YES | YES |
| CT260 (Mcsc) | 163 | 1-163 | YES | YES |
| CT262 | 256 | 1-256 | YES | YES |
| CT263 | 196 | 1-196 | YES | YES |
| CT273 | 188 | 1-188 | YES | YES |
| CT274 | 139 | 1-139 | YES | YES |
| CT276 | 193 | 1-193 | YES | YES |
| CT277 | 219 | 98-219 | YES | YES |
| CT283 | 698 | 1-698 | YES | YES |
| CT284 | 374 | 1-374 | YES | YES |
| CT286 | 853 | 1-853 | ------- | YES |
| CT289 | 337 | 1-337 | YES | YES |
| CT301 | 934 | 1-934 | YES | YES |
| CT324a | 303 | 184-303 | YES | YES |
| CT324 | 303 | 1-303 | YES | YES |
| CT325 | 138 | 1-138 | YES | YES |
| CT338 | 153 | 1-153 | YES | YES |
| CT339 | 509 | 91-286 | YES | YES |
| CT343 | 210 | 1-210 | YES | ------- |
| CT350 | 566 | 18-566 | YES | YES |
| CT351 | 697 | 1-697 | YES | YES |
| CT352 | 101 | 1-101 | YES | ------- |
| CT355 | 353 | 1-353 | YES | ------- |
| CT356 | 704 | 1-704 | YES | ------- |
| CT358 | 178 | 1-178 | YES | ------- |
| CT365 | 575 | 1-575 | YES | YES |
| CT372 | 442 | 1-442 | YES | YES |
| CT373 | 175 | 1-175 | YES | YES |
| CT377 | 46 | 1-46 | YES | ------- |
| CT379 | 447 | 1-447 | YES | YES |
| CT383a | 243 | 1-101 | YES | YES |
| CT383b | 243 | 155-243 | YES | YES |
| CT384 | 539 | 1-539 | YES | YES |
| CT385 | 111 | 1-111 | ------- | YES |
| CT389 | 408 | 1-408 | YES | YES |
| CT391 | 335 | 27-335 | YES | YES |
| CT398 | 254 | 1-254 | ------- | YES |
| CT418 | 335 | 1-335 | YES | YES |
| CT421 | 233 | 105-233 | YES | YES |
| CT423 | 369 | 90-369 | YES | ------- |
| CT429 | 329 | 1-329 | YES | YES |
| CT442 | 150 | 71-150 | YES | YES |
| CT456 (Tarp) | 1005 | 1-1005 | YES | YES |
| CT465 | 213 | 22-213 | YES | YES |
| CT469 | 178 | 1-178 | YES | YES |
| CT470 | 243 | 1-243 | YES | YES |
| CT471 | 200 | 26-200 | YES | YES |
| CT472 | 264 | 1-242 | YES | YES |
| CT473 (Lda3) | 104 | 1-104 | YES | YES |
| CT474 | 336 | 1-336 | YES | YES |
| CT476 | 321 | 21-321 | YES | YES |
| CT488 | 244 | 1-244 | YES | YES |
| CT504 | 288 | 1-288 | YES | YES |
| CT529a (Cap1) | 298 | 1-255 | YES | YES |
| CT529 (Cap1) | 298 | 1-298 | YES | YES |
| CT547 | 318 | 21-318 | YES | YES |
| CT548 | 194 | 20-194 | YES | YES |
| CT550 | 141 | 1-141 | YES | YES |
| CT556 | 159 | 1-101 | YES | YES |
| CT558 (LipA) | 311 | 1-311 | YES | ------- |
| CT559 (CdsJ) | 326 | 1-326 | YES | YES |
| CT560 | 278 | 1-278 | YES | YES |
| CT561 (CdsL) | 223 | 1-223 | YES | YES |
| CT562 (CdsR) | 306 | 1-306 | YES | ------- |
| CT563 (CdsS) | 94 | 1-94 | YES | YES |
| CT564 (CdsT) | 289 | 1-289 | YES | YES |
| CT565a | 147 | 1-110 | YES | YES |
| CT565 | 147 | 1-147 | YES | YES |
| CT566 | 330 | 20-330 | YES | YES |
| CT567 | 174 | 1-174 | YES | YES |
| CT568 | 151 | 41-151 | YES | ------- |
| CT569 | 109 | 1-109 | YES | YES |
| CT573 | 409 | 1-409 | ------- | YES |
| CT576 (Scc2) | 232 | 1-232 | YES | YES |
| CT577 | 119 | 1-119 | YES | YES |
| CT578a (CopB) | 487 | 1-254 | YES | YES |
| CT578b (CopB) | 487 | 299-487 | YES | ------- |
| CT579a (CopD) | 439 | 1-204 | YES | YES |
| CT579b (CopD) | 439 | 219-439 | YES | YES |
| CT580 | 327 | 1-327 | YES | YES |
| CT583 | 263 | 1-263 | YES | YES |
| CT584 | 183 | 1-183 | YES | YES |
| CT598 | 235 | 1-235 | YES | YES |
| CT602 | 130 | 1-130 | YES | YES |
| CT610 | 231 | 1-231 | YES | ------- |
| CT618 | 266 | 1-189 | YES | YES |
| CT620 | 838 | 1-838 | YES | YES |
| CT621 | 823 | 1-823 | YES | YES |
| CT622 | 647 | 1-647 | YES | YES |
| CT623 | 446 | 1-446 | YES | YES |
| CT632 | 529 | 1-529 | YES | YES |
| CT642 | 271 | 1-271 | ------- | YES |
| CT648 | 424 | 1-424 | YES | YES |
| CT663 | 133 | 1-183 | ------- | YES |
| CT664 (CdsD) | 829 | 1-829 | YES | YES |
| CT665 (CdsE) | 83 | 1-83 | YES | YES |
| CT666 (CdsF) | 83 | 1-83 | YES | YES |
| CT667 (CdsG) | 149 | 1-149 | ------- | YES |
| CT668 | 223 | 1-223 | YES | YES |
| CT669 (YscN) | 442 | 1-442 | YES | YES |
| CT670 | 168 | 1-168 | YES | YES |
| CT672 (CdsQ) | 373 | 1-373 | YES | YES |
| CT673 (Pkn5) | 490 | 1-490 | YES | YES |
| CT674 (CdsC) | 921 | 1-921 | YES | ------- |
| CT676 | 173 | 1-173 | YES | YES |
| CT677 (Rrf) | 179 | 1-179 | YES | YES |
| CT694 | 323 | 1-323 | YES | YES |
| CT695 | 398 | 1-398 | YES | YES |
| CT696 | 403 | 1-403 | YES | YES |
| CT700 | 441 | 16-441 | YES | YES |
| CT702 | 175 | 1-175 | YES | YES |
| CT711 | 767 | 1-767 | YES | YES |
| CT712 | 390 | 1-390 | YES | ------- |
| CT718 | 174 | 1-174 | YES | YES |
| CT724 | 174 | 1-174 | YES | YES |
| CT728 | 248 | 73-248 | ------- | YES |
| CT733 | 448 | 26-448 | YES | YES |
| CT734 | 221 | 1-221 | YES | YES |
| CT768 | 562 | 1-562 | YES | YES |
| CT779 | 229 | 1-229 | YES | YES |
| CT788 | 166 | 1-166 | YES | YES |
| CT795 | 163 | 13-163 | YES | YES |
| CT813 | 264 | 95-264 | ------- | YES |
| CT814 | 133 | 1-133 | YES | YES |
| CT824 | 974 | 1-974 | YES | YES |
| CT837 | 658 | 1-658 | YES | YES |
| CT845 | 92 | 1-92 | YES | YES |
| CT847 | 172 | 1-172 | YES | YES |
| CT848 | 168 | 1-168 | YES | YES |
| CT849 | 159 | 1-159 | YES | ------- |
| CT850 | 405 | 124-405 | YES | YES |
| CT858 (Cpaf) | 609 | 1-609 | YES | ------- |
| CT860 (Scc3) | 493 | 1-493 | YES | YES |
| CT861 (CopB2) | 506 | 1-506 | YES | ------- |
| CT862 (CopD2) | 198 | 1-198 | YES | YES |
| CT863 | 482 | 1-482 | YES | YES |
| CT867 (Chladub2) | 339 | 54-339 | YES | YES |
| CT868 (Chladub1) | 418 | 58-418 | YES | YES |

(a). *Chlamydia trachomatis* open reading frames are denoted by the nomenclature established for the sequenced genome of D/UW-3/CX. Common names of are marked with parenthesis. Abbreviations: *C. trachomatis* (CT) open reading frames (ORFs).

(b). ORF length obtained from STDGEN database (stdgen.northwestern.edu). Abbreviation: aa: amino acids

(c). Region of ORF cloned in this screen. Partial ORFs were designed to avoid large bilobed hydrophobic motifs that may hinder expression in yeast.

(d). The listed CT ORFs were subcloned into pGAD424 and subsequently transformed in to Mat***a*** yeast strain PJ69-4a. "-------" denote CT ORFs that were not cloned into the GAD vector.

(e). The listed CT ORFs were sublconed into pGBT9 and subsequently transformed in to Mat**α** yeast strain AH109. "-------" denote CT ORFs that were not cloned into the GBT9 vector.
