# Supplementary material for: The Chlamydia Type III Secretion System C-ring Engages a Chaperone-Effector Protein Complex
Source: PLoS Pathog. 2009 Sep 11;5(9):e1000579. doi: 10.1371/journal.ppat.1000579 (PMC2734247; doi:10.1371/journal.ppat.1000579)
Supplement: Table S2 — Summary of Y2H-based protein-protein interactions identified between chlamydial proteins (0.18 MB DOC) [file ppat.1000579.s002.doc]

Table S2: Summary of Y2H-based protein-protein interactions identified between chlamydial proteins

| ORFs ^a^ | MW (kDa) ^b^ | aa length ^c^ | Prot. Segment tested (aa) ^d^ | pI ^e^ | mRNA expression profile ^f^ | Proteomics analysis ^g^ | Description/function ^h^ | Pfam descriptions ^i^ | Species conservation ^j^ | Interacting partner ^k^ | Strength of interaction ^l^ |
| --- | --- | --- | --- | --- | --- | --- | --- | --- | --- | --- | --- |
| CT059 | 9.72 | 91 | 1-91 | 3.77 | 8hpi |  | ferrodoxin | beta-Grasp fold/Fer2 family | Tc, Cpn, Cca,Cab, Ct,Cf | CT229 | weak |
| CT060 (flhA) | 67.29 | 605 | 1-605 | 7.32 | 8hpi |  | flagellar biosynthesis and secretion protein | ClpB protein fold/ FHIPEP family | Tc, Cpn, Cca,Cab, Ct,Cf | CdsQ  (CT672) | weak |
| CT085 | 65.31 | 579 | 1-579 | 7.06 | 8hpi |  | Unknown function/ Contains ACR domains | Carboxylase/ UbiD family | Tc, Cpn, Cca,Cab, Ct,Cf | CdsQ  (CT672) | weak |
| CT088 (Scc1) | 16.18 | 146 | 1-146 | 6.77 | 8hpi | EB | Type Three Secretion chaperone protein | Tir chaperone protein family | Tc, Cpn, Cca,Cab, Ct,Cf | CT663 | strong |
| CT089 (CopN) | 45.09 | 421 | 1-421 | 4.87 | 16hpi |  | Type Three secretion target | low calcium response E (LcrE/SycD) | Tc, Cpn, Cca,Cab, Ct,Cf | Scc3  (Ct862) | moderate |
| CT119 (IncA) | 30.31 | 273 | 109-273 | 8.62 | 16hpi |  | Inclusion membrane protein/ inclusion fusion | IncA family | Cca,Cf,Ct | IncA  (CT119) | weak |
|  |  |  |  |  |  |  |  |  |  | CT226 | weak |
| CT161 | 27.64 | 246 | 1-246 | 9.2 | 16hpi |  | Unknown function |  | Ct | CT274 | weak |
| CT163 (Lda2) | 63.74 | 548 | 1-548 | 8.52 | 8hpi |  | Lipid droplet association | IQ calmodulin-binding motif | Ct, Tc | CT418 | weak |
| CT192 | 27.97 | 257 | 107-257 | 5.31 | 3hpi |  | Unknown function |  | Ct,Tc | CT283 | weak |
| CT223 | 29.47 | 270 | 86-270 | 7.97 | 8hpi | EB | Inclusion membrane protein | IncA family | Ct | CT223 | strong |
|  |  |  |  |  |  |  |  |  |  | CT229 | strong |
|  |  |  |  |  |  |  |  |  |  | CT700 | moderate |
| CT225 | 13.26 | 122 | 69-122 | 8.66 | 3hpi |  | Putative Inclusion membrane protein |  | Ct | Mcsc  (CT672) | strong |
| CT226 | 18.85 | 176 | 99-176 | 4.91 | 3hpi |  | Putative Inclusion membrane protein | IncA family | Ct, Tc | IncA  (CT119) | weak |
|  |  |  |  |  |  |  |  |  |  | CT700 | weak |
| CT229 | 23.42 | 215 | 92-215 | 8.42 | 1hpi |  | Inclusion membrane protein/Rab4 binding partner | IncA family | Ct, Tc | CT059 | weak |
|  |  |  |  |  |  |  |  |  |  | CT223 | Strong |
| CT260 (Mcsc) | 18.86 | 163 | 1-163 | 4.6 | 8hpi | EB | Unknown function | DUF1790 | Tc, Cpn, Cca,Cab, Ct,Cf | CT225 | strong |
|  |  |  |  |  |  |  |  |  |  | Mcsc  (CT260) | weak |
|  |  |  |  |  |  |  |  |  |  | Cap1  (CT529) | moderate |
|  |  |  |  |  |  |  |  |  |  | CT618 | strong |
|  |  |  |  |  |  |  |  |  |  | CdsQ  (CT672) | weak |
| CT274 | 15.59 | 139 | 1-139 | 4.45 | 8hpi |  | Unknown function | TPR domains | Tc, Cpn, Cca,Cab, Ct,Cf | CT161 | weak |
|  |  |  |  |  |  |  |  |  |  | CT668 | weak |
| CT276 | 21.35 | 193 | 1-193 | 4.27 | 8hpi |  | Unknown function | DUF583 | Tc, Cpn, Cca,Cab, Ct,Cf | CT276 | moderate |
|  |  |  |  |  |  |  |  |  |  | CT700 | weak |
| CT283 | 80.83 | 698 | 1-698 | 8.54 | 8hpi |  | Unknown function |  | Tc, Cpn, Cca,Cab, Ct,Cf | CT192 | weak |
| CT418 | 36.82 | 233 | 1-335 | 6.71 | 8hpi |  | GTP binding protein | OBG Fold | Tc, Cpn, Cca,Cab, Ct,Cf | Lda2  (CT163) | weak |
|  |  |  |  |  |  |  |  |  |  | CT700 | weak |
| CT472 | 30.16 | 264 | 1-242 | 5.12 | 8hpi | RB | Unknown function | OBG Fold, GPTase of unknown function, DUF155 | Tc, Cpn, Cca,Cab, Ct,Cf | CT677 | weak |
| CT473 (Lda3) | 11.72 | 104 | 1-104 | 9.49 | 1hpi |  | Lipid droplet association | DUF37 | Tc, Cpn, Cca,Cab, Ct,Cf | Lda3  (CT473) | moderate |
| CT529 (Cap1) | 30.98 | 298 | 1-255 | 9.83 | 1hpi |  | Inclusion memebrane protein |  | Tc, Cpn, Cca,Cab, Ct,Cf | Mcsc  (CT260) | strong |
| CT560 | 32.04 | 278 | 1-278 | 7.05 | 8hpi | EB | Unknown function |  | Tc, Cpn, Cca,Cab, Ct,Cf | CdsQ  (CT672) | moderate |
| CT561 (CdsL) | 24.78 | 223 | 1-223 | 5.82 | 16hpi | EB | Type Three Secretion Apparatus component/ ATPase inhibitor | FliH domain | Tc, Cpn, Cca,Cab, Ct,Cf | CdsQ  (CT672) | strong |
| CT563 (CdsS) | 10.56 | 94 | 1-94 | 9.36 | 16hpi |  | Type Three Secretion Apparatus component | Bacterial export proteins family 3 | Tc, Cpn, Cca,Cab, Ct,Cf | CdsQ  (CT672) | weak |
| CT564 (CdsT) | 32.33 | 289 | 1-289 | 7.12 | 8hpi |  | Type Three Secretion Apparatus component | Bacterial export proteins family 1 | Tc, Cpn, Cca,Cab, Ct,Cf | CT648 | weak |
|  |  |  |  |  |  |  |  |  |  | CdsQ  (CT672) | weak |
| CT565 | 15.96 | 147 | 1-147 | 9.37 | 8hpi |  | Unknown function |  | Tc, Cpn, Cca,Cab, Ct,Cf | CT565 | weak |
| CT567 | 20.23 | 174 | 1-174 | 10.4 | 8hpi |  | Unknown function | DUF1494 | Tc, Cpn, Cca,Cab, Ct,Cf | CdsQ  (CT672) | weak |
| CT576 (Scc2) | 26.01 | 232 | 1-232 | 9.58 | 16hpi | EB | Type Three Secretion chaperone protein similar to lcrH | TPR family 3  SycD | Tc, Cpn, Cca,Cab, Ct,Cf | CopB  (CT578) | moderate |
|  |  |  |  |  |  |  |  |  |  | CopD  (CT578) | moderate |
| CT578 (CopB) | 50.21 | 487 | 1-254 | 9.74 | 24hpi | EB | Target of TTSS |  | Tc, Cpn, Cca,Cab, Ct,Cf | Scc2  (CT576) | moderate |
| CT579 (CopD) | 44.01 | 439 | 1-204 | 10 | 24hpi | EB | Target of TTSS |  | Tc, Cpn, Cca,Cab, Ct,Cf | Scc2  (CT576) | moderate |
| CT584 | 21.13 | 183 | 1-183 | 5.74 | 16hpi | EB,RB | Unknown function |  | Tc, Cpn, Cca,Cab, Ct,Cf | CdsF  (CT666) | moderate |
| CT618 | 27.92 | 266 | 1-189 | 5.23 | 8hpi | RB | Inclusion membrane protein |  | Tc, Cpn, Cca,Cab, Ct,Cf | Mcsc  (CT260) | strong |
| CT621 | 92.7 | 632 | 1-823 | 4.76 | 8hpi | RB | Unknown function | DUF582 | Tc, Cpn, Cca,Cab, Ct,Cf | CdsN  (CT669) | weak |
| CT648 | 47.83 | 424 | 1-424 | 7.56 | 1hpi |  | Unknown function |  | Tc, Cpn, Cca,Cab, Ct,Cf | CdsT  (CT564) | moderate |
|  |  |  |  |  |  |  |  |  |  | CT676 | moderate |
| CT663 | 14.66 | 133 | 1-133 | 4.08 | 8hpi |  | Type Three Secretion chaperone protein | Tir chaperone protein family | Tc, Cpn, Cca,Cab, Ct,Cf | Scc1  (CT088) | strong |
| CT665 (CdsE) | 9.28 | 83 | 1-83 | 9.92 | 8hpi |  | Type Three Secretion chaperone protein |  | Tc, Cpn, Cca,Cab, Ct,Cf | CdsG  (CT667) | strong |
| CT666 (CdsF) | 9.14 | 83 | 1-83 | 4.48 | 8hpi | EB | Type Three Secretion Apparatus needle |  | Tc, Cpn, Cca,Cab, Ct,Cf | CT584 | moderate |
|  |  |  |  |  |  |  |  |  |  | CdsG  (CT667) | moderate |
| CT667 (CdsG) | 16.45 | 149 | 1-149 | 4.72 | 8hpi |  | Type Three Secretion chaperone protein | TPR family 2 | Tc, Cpn, Cca,Cab, Ct,Cf | CdsE  (CT665) | strong |
|  |  |  |  |  |  |  |  |  |  | CdsF  (CT666) | moderate |
| CT668 | 24.43 | 223 | 1-223 | 4.43 | 8hpi |  | Unknown function |  | Tc, Cpn, Cca,Cab, Ct,Cf | CT274 | weak |
|  |  |  |  |  |  |  |  |  |  | CT837 | moderate |
| CT669 (CdsN) | 48.19 | 442 | 1-442 | 5.63 | 16hpi | EB | Type Three Secretion Apparatus | ATP synthase alpha/beta family | Tc, Cpn, Cca,Cab, Ct,Cf | CT621 | weak |
| CT672 (CdsQ) | 41.39 | 373 | 1-373 | 4.45 | 16hpi | EB | Type Three Secretion Apparatus component | SpoA, YscQ, Spa33, FliM/N | Tc, Cpn, Cca,Cab, Ct,Cf | FlhA  (CT060) | weak |
|  |  |  |  |  |  |  |  |  |  | CT085 | weak |
|  |  |  |  |  |  |  |  |  |  | Mcsc  (CT260) | weak |
|  |  |  |  |  |  |  |  |  |  | CT560 | moderate |
|  |  |  |  |  |  |  |  |  |  | CdsL  (CT561) | strong |
|  |  |  |  |  |  |  |  |  |  | CdsS  (CT563) | weak |
|  |  |  |  |  |  |  |  |  |  | CdsT  (CT564) | weak |
|  |  |  |  |  |  |  |  |  |  | CT567 | weak |
|  |  |  |  |  |  |  |  |  |  | CT676 | weak |
|  |  |  |  |  |  |  |  |  |  | CT677 | weak |
|  |  |  |  |  |  |  |  |  |  | CT824 | weak |
| Ct676 | 19.84 | 173 | 1-173 | 5.67 | 8hpi | EB | Unknown function | Uvrb/UvrC motif | Tc, Cpn, Cca,Cab, Ct,Cf | CT648 | moderate |
|  |  |  |  |  |  |  |  |  |  | CdsQ  (CT672) | weak |
| CT677 | 20.07 | 179 | 1-179 | 9.08 | 3hpi | EB | ribosomal recycling factor | RRF domain | Tc, Cpn, Cca,Cab, Ct,Cf | CT472 | weak |
|  |  |  |  |  |  |  |  |  |  | CdsQ  (CT672) | weak |
| CT700 | 50.41 | 441 | 16-441 | 8.44 | 8hpi |  | Unknown function/ TPR | TPR family 2 | Tc, Cpn, Cca,Cab, Ct,Cf | CT226 | weak |
|  |  |  |  |  |  |  |  |  |  | CT276 | weak |
|  |  |  |  |  |  |  |  |  |  | CT418 | weak |
|  |  |  |  |  |  |  |  |  |  | CT824 | weak |
| CT712 | 43.99 | 390 | 1-390 | 5.07 | 16hpi |  | Unknown function | DUF582 | Tc, Cpn, Cca,Cab, Ct,Cf | CT768 | weak |
| CT768 | 64.26 | 562 | 1-562 | 5.21 | 8hpi | EB | Unknown function |  | Tc, Cpn, Cca,Cab, Ct,Cf | CT712 | weak |
| CT824 | 109.3 | 974 | 1-974 | 5.18 | 8hpi |  | putative metalloprotease | Metallopeptidase family M16 | Tc, Cpn, Cca,Cab, Ct,Cf | CdsQ  (CT672) | weak |
|  |  |  |  |  |  |  |  |  |  | CT700 | weak |
| CT837 | 76.49 | 658 | 1-658 | 6.06 | 8hpi | EB | Unknown function |  | Tc, Cpn, Cca,Cab, Ct,Cf | CT668 | moderate |
| CT862 (Scc3) | 23.29 | 198 | 1-198 | 6.71 | 16hpi |  | Type Three Secretion chaperone protein | TPR family 3  SycN | Tc, Cpn, Cca,Cab, Ct,Cf | CopN  (CT089) | strong |

(a). *Chlamydia trachomatis* open reading frame are denoted by the nomenclature established in Stephens RS, Kalman S, Lammel C, Fan J, Marathe R, et al. (1998) Science 282: 754-759.. Common names of CT ORFs are marked with parenthesis. Abbreviations: *Chlamydia trachomatis* (CT) open reading frames (ORFs).

(b). CT ORF gene lengths obtained from STDGEN Database (stdgen.northwestern.edu).

(c). CT ORF amino acid length based on STDGEN Database (stdgen.northwestern.edu). Abbreviations: amino acid (aa)

(d). Region of CT ORF incorporated in yeast two-hybrid screen.

(e). Calculated isoelectric point (pI) of CT ORFs used in this study obtained from STDGEN Database (stdgen.northwestern.edu).

(f). Temporal expression profile as established by Belland et al. (2003) PNAS 100:8478-8483.

(g). Proteomic profile derived from Skipp P. RJ, O'Connor C.D. and Clarke I.N. (2005) Proteomics 5: 1558-1573.

(h.) Description of CT ORF's known or putative function obtained from STDGEN database (stdgen.northwestern.edu).

(i). Similarities to protein families, motifs or domains as determined by using Pfam (pfam.sanger.ac.uk).

(j). Homology searches of CT ORF against other chlamydial species (Comprehensive Microbial Resource Blast analysis tool - tigrblast.tigr.org/cmr-blast/). Abbreviations: *Chlamydia muridarum* (Tc), *Chlamydia pneumoniae* (Cpn), *Chlamydophila caviae* (Cca), *Chlamydia abortus* (Cab), *Chlamydia trachomatis* (Ct), *Chlamydophila felis* (Cf).

(k). List of interacting CT ORFs as determined by yeast two-hybrid analysis.

(l). Relative strength of interacting CT ORFs was assessed based on observed growth of diploid reporter yeast strains on synthetic media lacking histidine or adenine. Note: The strength of interactions is arbitrary as it influenced by protein expression levels, toxicity and efficiency of translocation of fusion proteins to the nucleus.
