# Supplementary material for: The Chlamydia Type III Secretion System C-ring Engages a Chaperone-Effector Protein Complex
Source: PLoS Pathog. 2009 Sep 11;5(9):e1000579. doi: 10.1371/journal.ppat.1000579 (PMC2734247; doi:10.1371/journal.ppat.1000579)
Supplement: Table S3 — Strains, plasmids and primers (0.06 MB DOC) [file ppat.1000579.s003.doc]

Table S3: Strains, plasmids and primers

**Yeast Strains**

| **Strain** | **Characteristics** | **Source** |
| --- | --- | --- |
| PJ69-4a | MATα *trp1-901 leu2-3, 112 ura3-53 his3-200 gal4Δ gal80Δ GAL2-ADE LYS2::GAL1-HIS3 met::GAL7-lacz* | Laboratory stock |
| AH109 | MATa *trp1-901 leu2-3, 112 ura3-53 his3-200 gal4Δ gal80Δ GAL2-ADE LYS2::GAL1-HIS3 met::GAL7-lacz* | Clontech |

Bacterial Strains

| **Strain** | **Characteristics** | **Source** |
| --- | --- | --- |
| XL1-Blue | *recA1 endA1 gyrA96 thi-1 hsdR17 supE44 relA1 lac [F´ proAB lacIqZΔM15 Tn10 (Tetr)]* | Stratagene |
| BL21-DE3 | B F– *dcm ompT hsdS*(rB– mB–) *gal λ*(DE3) | Stratagene |

Plasmids

| **Strain** | **Description** | **Source** |
| --- | --- | --- |
| pGAD424 | N terminal *GAL4_AD_* fusion, *LEU2,* Amp-r, 2µ vector | Clontech |
| pGBT9 | N terminal *GAL4_DBD_* fusion, *TRP1*, Amp-r, 2µ vector | Clontech |
| pET15b | N terminal 6x His Tag, Amp-r, pBR322 origin | Novagene |
| pET24d | N terminal T7-Tag, 6x His C-terminal Tag, Kan-r, f1 origin | Novagene |
| pET24d-CT618 | CT618 (aa1-189) cloned into pET24d | This study |
| pET15b-Mcsc^a^ | Full length Mcsc cloned into pET15b | This study |
| pET24d-Mcsc/Cap1 | *MCSC* and *CAP1* were cloned into pET24d along with a stop codon and ribosomal binding site interposed between the genes | This study |
| pET24d-Mcsc/CT618 | *MCSC* and *CT618* were cloned into pET24d along with a stop codon and ribosomal binding site interposed between the genes | This study |
| pGEX-4T-1 | N terminal GST Tag, Amp-r, pBR322 origin | GE Healthcare |
| pGST-Mcsc | Mcsc cloned into pGEX-4T-1 | This Study |
| pET15b-Mcsc L15A | Residue L15 of Mcsc was mutated to an alanine | This study |
| pET15b-Mcsc 3A | Amino acid residues I31, I33 and V35 of Mcsc were mutated to alanines. | This study |
| pET15b-Mcsc 3G | Amino acid residues I31, I33 and V35 of Mcsc were mutated to glycine. | This study |
| pGST-CdsQ | CdsQ cloned into pGEX-4T-1 | This Study |

Primers

| **Primer Name** | **N-terminal Sequence^b^** | **C-terminal Seguence^b^** |
| --- | --- | --- |
| pGAD424 | CTATTCGATGATGAAGATACCCCACCAAACCCAAAAAAAGAGATCCCGGATTCTAGAACTAGTATG | GTTTTTCAGTATCTACGATTCATAGATCTCTGCAGGTCGACGTTCTCCTTTACTCATAAGCTT |
| pGAD424-CT056 | CTATTCGATGATGAAGATACCCCACCAAACCCAAAAAAAGAGATCATGACACTCCCTTCCGAAGC | GTTTTTCAGTATCTACGATTCATAGATCTCTGCAGGTCGACGAGGAAGAAGAAGAACTGCAGTG |
| pGAD424-CT082 | CTATTCGATGATGAAGATACCCCACCAAACCCAAAAAAAGAGATCATGTCAATTTCTGGAAGTGG | GTTTTTCAGTATCTACGATTCATAGATCTCTGCAGGTCGACGATCGCCTCCTGCATCCTCTGTTTC |
| pGAD424-CT085 | CTATTCGATGATGAAGATACCCCACCAAACCCAAAAAAAGAGATCGTGTTTTCCTTACGATCTTTAG | GTTTTTCAGTATCTACGATTCATAGATCTCTGCAGGTCGACGGAAATAGCTATGCCAGCGAG |
| pGAD424-CT090 | CTATTCGATGATGAAGATACCCCACCAAACCCAAAAAAAGAGATCATGAACAAGCTACTCAACTTTG | GTTTTTCAGTATCTACGATTCATAGATCTCTGCAGGTCGACGAATTTGAATTCTTCCCAAAGG |
| pGAD424-CT091 | CTATTCGATGATGAAGATACCCCACCAAACCCAAAAAAAGAGATCATGGGCGAAAAAACAGAAAAG | GTTTTTCAGTATCTACGATTCATAGATCTCTGCAGGTCGACGATTATCGAATTGGTTAATGTT |
| pGAD424-CT562 | CTATTCGATGATGAAGATACCCCACCAAACCCAAAAAAAGAGATCATGCGATTGATTGTTCGAATT | GTTTTTCAGTATCTACGATTCATAGATCTCTGCAGGTCGACGAAAGCTAATCATCAACCCTTC |
| pGAD424-CT671 | CTATTCGATGATGAAGATACCCCACCAAACCCAAAAAAAGAGATCATGGAATTAAATAAAACTTCG | GTTTTTCAGTATCTACGATTCATAGATCTCTGCAGGTCGACGTATATGAGCTTCTTCTACTTTC |
| pGAD424-CT719 | CTATTCGATGATGAAGATACCCCACCAAACCCAAAAAAAGAGATCATGCCGGTGCTACCTCGC | GTTTTTCAGTATCTACGATTCATAGATCTCTGCAGGTCGACGTGTTCTTAACTTTAACAC |
| pGBT9 | TCATCGGAAGAGAGTAGTAACAAAGGTCAAAGACAGTTGACTGTATCGCCGGATTCTAGAACTAGTATG | AATCATAAGAAATTCGCCCGGAATTAGCTTGGCTGCAGGTCGACGTTCTCCTTTACTCATAAGCTT |
| pET24d-Mcsc | TATACTTTAACGTCAAGG | CTCGAATTCGGATCCTATATCTCCTTCCTAAGGCTCTAGCTGATCGGATTGG |
| pET24d-Cap1 | CAAATGGGTCGGATCCTTATGGCTTCTATATGCGGACG | GTGCTCGAGTGCGGCCGCCTCTCCCGGCACTTCCAGTAA |
| pET24d-CT618 | CAAATGGGTCGGATCCTTATGGCAGCAACGGTACCCATAGC | GTGCTCGAGTGCGGCCGCCTGTTTGAGGTTCCCGAGAAC |
| Mcsc L15A^c^ | CTTACAAAATTCGCGACCCATGCTCAGCTAGAACC | CTAGCTGAGCATGGGTCGCGAATTTTGTAAGATTATTGTG |
| Mcsc 3A^c^ | GAGAAAGCGGCTTAGCCTATGCCACAGCCCCTGCGGGAGAACATG | GTTCTCCCGCAGGGGCTGTGGCATAGGCTAAGCCGCTTTCTCGTTC |
| Mcsc 3G^c^ | GAGAAAGCGGCTTAGGCTATGGCACAGGCCCTGCGGGAGAACATG | GTTCTCCCGCAGGGCCTGTGCCATAGCCTAAGCCGCTTTCTCGTTC |

(a). Creation of pET15b was done in the laboratory of Dr. Pei Zhou, Duke University.

(b). All primers shown in 5’ to 3’ orientation.

(c). Underlined codons denote sites mutated in *MCSC*.
