# Supplementary figures and images for: The Chlamydia Type III Secretion System C-ring Engages a Chaperone-Effector Protein Complex
Source: PLoS Pathog. 2009 Sep 11;5(9):e1000579. doi: 10.1371/journal.ppat.1000579 (PMC2734247; doi:10.1371/journal.ppat.1000579)

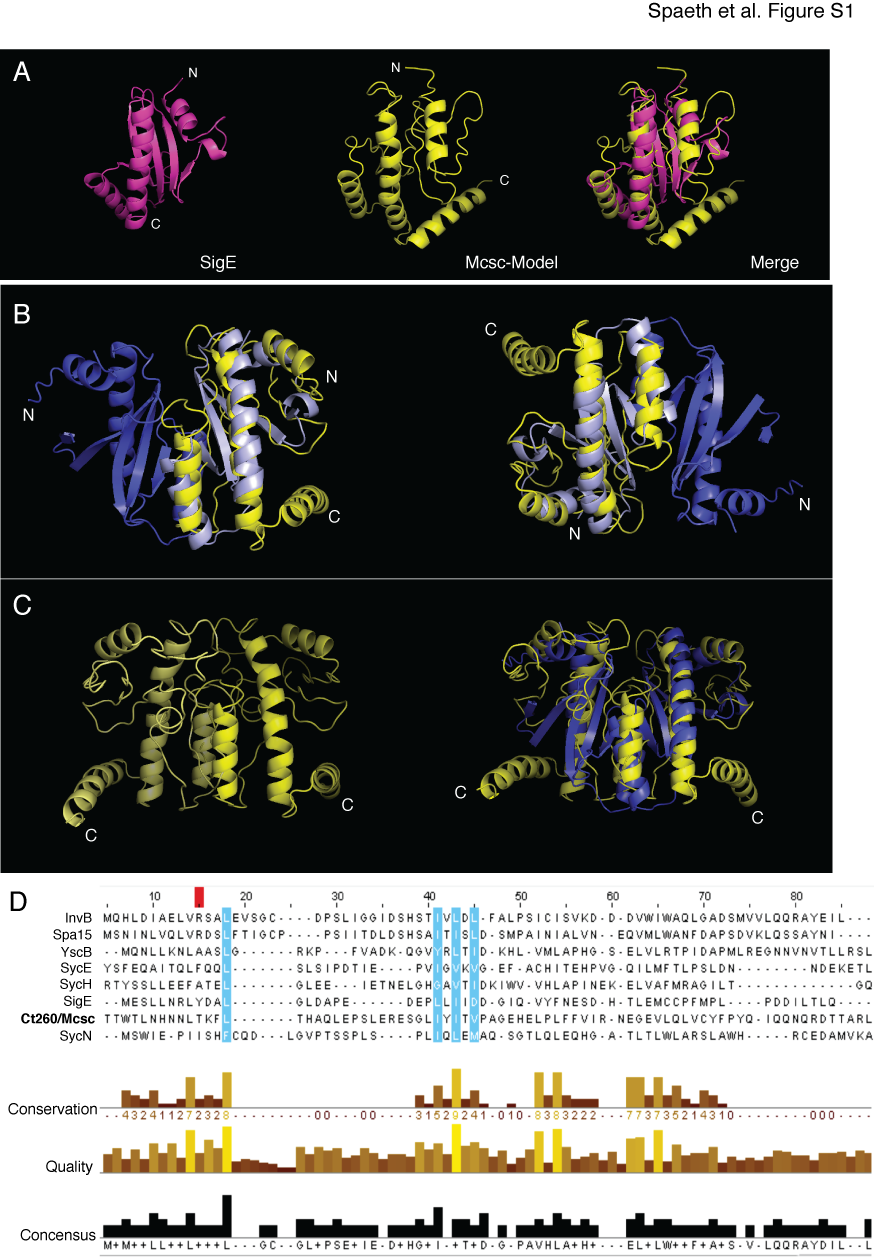

Supplement: Figure S1 — Mcsc structural models. A–C. Predicted three dimensional structure of Mcsc. A putative 3D model of Mcsc (yellow) was generated with the neural network folding prediction program I-TASSER (http://zhang.bioinformatics.ku.edu/I-TASSER/) [1]. Modeling of Mcsc and SigE structure were performed with PyMOL (http://www.pymol.org/). Overlay of Mcsc monomers (A) on SigE (magenta) monomers and dimers (blue) (B), and a model of a Mcsc dimer (C). D. Conservation among amino terminal residues in Class I T3S chaperones required for effector protein binding. Conserved amino acids (blue) in Mcsc align with α-helix and β-strand 1 as assessed by secondary structure predictions performed with PSIPRED (http://bioinf.cs.ucl.ac.uk/psipred/). (3.34 MB TIF) [file ppat.1000579.s004.tif]
